# Integrative network pharmacology, molecular dynamics simulation, and single-cell RNA sequencing strategies reveal the multi-target mechanisms of oridonin against cervical cancer

**DOI:** 10.3389/fphar.2026.1788376

**Published:** 2026-06-17

**Authors:** Min Xu, Tao Lu, Qing LV, Kaiju Mo, Yanjie Liu

**Affiliations:** 1 Department of Pathology, Guizhou Medical University, Guiyang, Guizhou, China; 2 Department of Pathology, Affiliated Hospital of Guizhou Medical University, Guiyang, Guizhou, China; 3 Department of Pathology, Sandu Shui Autonomous County People’s Hospital, Sandu, Guizhou, China; 4 Department of Pathology, Liupanshui Municipal People’s Hospital, Liupanshui, China; 5 Department of Pathology, Pathology Morphology and Molecular Laboratory, Affiliated Hospital of Guizhou Medical University, Guiyang, China

**Keywords:** cervical cancer, epithelial–mesenchymal transition, network pharmacology, oridonin, single-cell RNA sequencing

## Abstract

**Introduction:**

Cervical cancer remains a major threat to women’s health worldwide, and effective therapeutic strategies with clear molecular mechanisms are still needed. Oridonin (Ori), a natural diterpenoid compound, has shown antitumor activity in multiple malignancies; however, its regulatory mechanisms in cervical cancer, particularly at the single-cell level, remain incompletely understood.

**Methods:**

In this study, an integrative strategy combining network pharmacology, bulk transcriptomic analyses, single-cell RNA sequencing (scRNA-seq), molecular docking, molecular dynamics simulations, and *in vitro* experiments was employed to systematically investigate the pharmacological effects and mechanisms of oridonin in cervical cancer. Differentially expressed genes were identified from multiple Gene Expression Omnibus datasets, and core targets were screened through protein–protein interaction analysis. Immune infiltration patterns were evaluated using CIBERSORT, while cell-type–specific pathway activities were inferred based on scRNA-seq data. The predicted mechanisms were further validated by molecular simulations and functional assays in cervical cancer cell lines.

**Results:**

Network pharmacology and transcriptomic analyses identified 25 core EMT-related targets of oridonin, with enrichment primarily in the PI3K/AKT signaling pathway. Immune infiltration analysis revealed that oridonin-associated targets were closely correlated with macrophages and T-cell subsets. Single-cell analysis demonstrated that PI3K/AKT and EMT-related pathways were predominantly enriched in epithelial tumor cells, suggesting cell-type–specific regulatory effects. Molecular docking and dynamics simulations indicated favorable binding potential between oridonin and AKT1. *In vitro* experiments confirmed that oridonin significantly inhibited cervical cancer cell proliferation, migration, and invasion, induced apoptosis, and suppressed epithelial–mesenchymal transition progression via downregulation of the PI3K/AKT pathway.

**Conclusion:**

This study provides a systematic and multi-level characterization of the antitumor mechanisms of oridonin in cervical cancer. By integrating network pharmacology with single-cell transcriptomic analysis, our findings highlight the cell-type–specific modulation of the PI3K/AKT–EMT axis by oridonin, offering mechanistic insights and establishing a theoretical pharmacological basis that warrants future *in vivo* preclinical evaluation.

## 1 Introduction

Cervical cancer remains a major female malignancy with significant regional disparities despite advances in screening and prevention (Sung et al., 2021). Persistent infection with high-risk human papillomavirus (HPV) drives cervical epithelial cells from dysplasia to invasive carcinoma by disrupting tumor suppressors like p53 and pRb, impairing cell-cycle control, and providing sustained oncogenic stimulation (Moody and Laimins, 2010; Doorbar et al., 2012; Schiffman and Wentzensen, 2013). Surgery, radiotherapy, and chemotherapy are standard treatments, but their efficacy at recurrent or metastatic stages is limited by tumor heterogeneity, immunosuppressive microenvironments, and drug resistance (Tewari et al., 2014; Coleman et al., 2021; Colombo et al., 2021). High-throughput sequencing has revealed subtype-specific genetic alterations and pathway activations, offering insights for precision therapy (Ojesina et al., 2014; Hyeon et al., 2025). Among the various histological subtypes, cervical squamous cell carcinoma and endocervical adenocarcinoma (CESC) constitute the vast majority of cases and serve as the primary focus for such targeted transcriptomic profiling.

In CESC–associated signaling networks, aberrant activation of the PI3K/Akt pathway is common and is correlated with tumor progression and clinical prognosis (Tornesello et al., 2014; Fruman et al., 2017; Wang et al., 2024). Even in some cases with atypical evidence of HPV infection, this pathway may still promote tumor growth. Meanwhile, epithelial–mesenchymal transition (EMT) is closely associated with cervical cancer invasion, metastasis, and therapeutic resistance (Qureshi et al., 2015). Studies have shown that PI3K/Akt can participate in the initiation and maintenance of EMT by regulating molecules such as GSK-3β and Snail, thereby exerting synergistic effects during malignant tumor progression (Zhao et al., 2021).

Oridonin (Ori) is a diterpenoid compound derived from the traditional medicinal plant *Isodon rubescens*. Previous studies have demonstrated that this compound is capable of inhibiting cell proliferation, inducing cell death, and modulating inflammatory and oxidative stress responses in multiple tumor models (Li et al., 2021). Some experimental studies suggest that Ori may affect PI3K/Akt-related signaling activity; for example, in HeLa cells, it can induce apoptosis accompanied by alterations in PI3K/Akt signaling (Hu et al., 2007), and in melanoma and nasopharyngeal carcinoma models, inhibition of the PI3K/Akt/GSK-3β or AKT/STAT3 pathways has also been observed together with attenuation of EMT phenotypes (Li et al., 2018; Liu et al., 2021). However, these findings are mostly derived from single models or observations under specific conditions, and more systematic evidence is still lacking as to whether Ori exerts its effects in cervical cancer through PI3K/Akt–EMT–related mechanisms.

In recent years, network pharmacology has provided tools for elucidating the potential targets of natural products and for predicting the molecular pathways that may be involved at a systems level (Berger and Iyengar, 2009; Hopkins, 2008; Huang et al., 2014). Integration with public transcriptomic and clinical data facilitates evaluation of the relevance of these predictions across larger sample cohorts (Catalano et al., 2025). In addition, the application of single-cell transcriptomic technologies has enabled researchers to observe tumor heterogeneity and microenvironmental composition at the cellular scale (Cao et al., 2023; Fan et al., 2023; Lin et al., 2023; Wang et al., 2025). On this basis, combining multi-level computational analyses with *in vitro* functional experiments (Zhou et al., 2025) helps to more comprehensively evaluate the biological effects of Ori in cervical cancer (Supplementary Figure S1). Therefore, this study hypothesizes that oridonin may exert multi-targeted anti-cervical cancer effects by inhibiting the PI3K/AKT signaling pathway, reversing the EMT process, and modulating the tumor immune microenvironment.

## 2 Materials and methods

### 2.1 Reagents and materials

All reagents and materials used in this study are listed in Tables 1, 2.

**TABLE 1:** Main reagents used in this study.

Reagents	Manufacturer	Cat. no.
MEM	Wuhan procell life science & technology Co, Ltd	PM150410
ECL	Abbkine	P10100
Western BlotPrimary antibody diluent	Wuhan boster biological technology Co, Ltd	20F27C17
Rapid electrophoresis solution & transfer solution	Wuhan servicebio technology Co, Ltd	G2081-15
Crystal violet	Beijing Solarbio science & technology Co,Ltd	G1063
Apoptosis kit	MULITISCIENCES	AT105
CCK-8	Wuhan servicebio technology Co, Ltd	BMU106
PBS	Wuhan servicebio technology Co, Ltd	G4202
DMSO	Beijing Solarbio science & technology Co., Ltd	D8371
TBST	Wuhan servicebio technology Co, Ltd	G0004-1L
Gel rapid preparation kit	Wuhan servicebio technology Co, Ltd	PN3011
4% paraformaldehyde	Wuhan servicebio technology Co, Ltd	BL539A
FBS	Vincent biotechnology (nanjing) Co, Ltd	086–150
EDU	APEXBIO (USA)	K1078
Matrigel	Mojisheng biology	0,827,045

**TABLE 2:** Primary antibodies used in this study.

Antibodies	Cat. no.	Species	Companies	Dulution
BAX	YT0455	Rabbit	Immunoway	1:1000
Bcl-2	BF9103	Rabbit	Affinity biosciences	1:500
Caspase-3	F1080	Rabbit	Selleck	1:3000
Cleaved Caspase-3	F0135	Rabbit	Selleck	1:600
AKT	F0004	Rabbit	Selleck	1:600
p-AKT	ET1607-73	Rabbit	Huaan biotechnology	1:5000
PI3K	R22768	Rabbit	Zenbio	1:10,000
p-PI3K	341,468	Rabbit	Zenbio	1:800
E-Cadherin	1238-1-AP	Rabbit	Proteintech	1:2000
N-Cadherin	22018-1-AP	Rabbit	Proteintech	1:4000
Snail	340,942	Rabbit	Zenbio	1:6000
Vimentin	10366-1-AP	Rabbit	Proteintech	1:5000
GAPDH	81640-5-RR	Rabbit	Proteintech	1:15,000

### 2.2 Identification of Ori targets and functional enrichment analysis

The SMILES and two-dimensional SDF structural files of Ori were retrieved from PubChem for target prediction. Potential targets of Ori were predicted using multiple platforms based on its chemical structure. In SwissTargetPrediction, targets with a probability score >0.1 were retained to ensure high-confidence predictions. In PharmMapper, the top 300 targets were obtained via pharmacophore mapping, and those with a normalized fit score (Z-score) > 0.5 were selected. Predictions in TargetNet were conducted using default parameters, and targets with a probability >0.5 were retained. Experimentally validated or literature-supported targets were collected from the HERB database without additional thresholds.

All predicted targets were integrated and duplicates removed. Targets were standardized using UniProt (*Homo sapiens*) to obtain candidate targets. GO and KEGG enrichment analyses were performed using clusterProfiler in R (Yu et al., 2012), and results with adjusted P values <0.05 were considered statistically significant.

### 2.3 Data acquisition and standardization

Cervical cancer–related genes were retrieved from the GeneCards database using the keyword “cervical cancer,” and genes with a Relevance Score greater than 20 were selected to construct a disease-related gene set (Supplementary Material 1) to optimally balance analytical sensitivity and specificity. Transcriptomic data were obtained from the NCBI Gene Expression Omnibus (GEO) database, selecting datasets relevant to cervical cancer: GSE7803, GSE13080, and GSE9750. Specifically, GSE7803 included 28 cervical cancer tissue samples and 10 non-tumor tissue samples, GSE13080 included 25 tumor tissue samples and 10 normal tissue samples, and GSE9750 included 32 tumor tissue samples and 24 non-tumor tissue samples, which were used as an external validation set. To reduce technical differences between datasets, the gene expression matrices of GSE7803 and GSE13080 were merged. Surrogate variable analysis (SVA) was applied to estimate potential confounding factors, and batch effects were corrected using the ComBat algorithm. The data distribution before and after correction was assessed using principal component analysis (PCA). The epithelial–mesenchymal transition (EMT)–related gene set was obtained from the dbEMT2.0 database (https://bioinfo-minzhao.org/dbemt/) (Zhao et al., 2015) for subsequent EMT-related analyses.

### 2.4 Differential gene expression analysis

Transcriptomic data were analyzed using the limma package (Ritchie et al., 2015). Differentially expressed genes (DEGs) were identified with thresholds of an adjusted P-value (FDR) < 0.05 and an absolute log2 fold change (|log2*FC*|) > 0.585 (corresponding to a 1.5-fold change). The results were visualized using the ggplot2 package.

### 2.5 Weighted gene co-expression network analysis

WGCNA was applied to identify gene co-expression modules associated with cervical cancer (Langfelder and Horvath, 2008). Prior to network construction, hierarchical clustering was conducted to evaluate sample quality and exclude outlier samples. The soft-thresholding power was determined according to the scale-free topology criterion, ensuring that the topology fit index (R^2^) exceeded 0.8. Subsequently, a topological overlap matrix (TOM) was constructed, and modules were identified using the dynamic tree cut algorithm, with a minimum module size of 60 genes and a module merging threshold height of 0.25. The relationships between modules and clinical phenotypes were assessed by calculating Pearson correlation coefficients between module eigengenes (MEs) and phenotypes, and modules with an absolute correlation coefficient greater than 0.5 and *P* < 0.05 were defined as key modules. Within the key modules, hub genes were screened based on module membership (kME >0.8), and module membership (MM) and gene significance (GS) were calculated for further analysis.

### 2.6 Disease target identification

To obtain a highly precise and robust set of pathogenic genes associated with cervical cancer, differentially expressed genes (DEGs) were first intersected with hub genes from key WGCNA modules. Given the high-dimensional nature and potential multicollinearity of this initial candidate pool, the Least Absolute Shrinkage and Selection Operator (LASSO) regression algorithm was applied for rigorous feature selection and dimensionality reduction (Shi et al., 2024). By employing an L1 penalty via the R package, the coefficients of highly correlated, redundant variables were computationally shrunk to zero. This penalized regression approach effectively mitigated the target inflation inherent in network pharmacology, allowing for the extraction of a sparse, non-redundant subset of core pathogenic targets essential for high-specificity downstream analyses, such as molecular docking and single-cell mapping. Subsequently, the expression profiles of these optimally selected genes were compared between control and tumor groups using boxplots, and their stability was externally validated using the GSE9750 dataset. Finally, these filtered targets were integrated with known cervical cancer-related genes retrieved from the GeneCards database to construct a high-confidence disease target gene network for subsequent topological analyses.

### 2.7 Screening of therapeutic targets and PPI analysis

Disease target genes were intersected with candidate drug targets of Ori to identify potential therapeutic targets of Ori against cervical cancer. A protein–protein interaction (PPI) network was constructed using the STRING database (Szklarczyk et al., 2021), with the species restricted to *Homo sapiens* and the interaction confidence score set to ≥0.7. Cytoscape (version 3.10.0) was used for network visualization and topological analysis, and two rounds of node screening were performed using the CytoNCA plugin (Tang et al., 2015). The screening criteria included betweenness centrality (BC), degree centrality (DC), closeness centrality (CC), eigenvector centrality (EC), local average connectivity (LAC), and network centrality (NC), with all parameters required to exceed their respective median values. Ultimately, core genes within the PPI network were identified and compared with an epithelial–mesenchymal transition (EMT)–related gene set to determine the key therapeutic targets through which Ori regulates the EMT process.

### 2.8 Functional enrichment analysis

Functional enrichment analysis of key therapeutic target genes was conducted using the clusterProfiler package, including Gene Ontology (GO) analysis and Kyoto Encyclopedia of Genes and Genomes (KEGG) pathway analysis. GO analysis encompassed biological processes (BP), molecular functions (MF), and cellular components (CC). A P value <0.05 was set as the threshold for statistical significance.

### 2.9 Immune infiltration analysis

The CIBERSORT algorithm and its LM22 signature gene matrix (Newman et al., 2015) were used to estimate the immune cell composition of samples from the GSE7803 and GSE13080 datasets, and to calculate the relative proportions of 22 immune cell subtypes in each sample. Immune infiltration results were presented as heatmaps, and boxplots were used to compare differences in the proportions of each immune cell subtype between the control group and the cervical cancer group. The corrplot package was applied to analyze and visualize correlations among different immune cell subtypes. o further investigate the potential immunoregulatory effects of Ori against cervical cancer, Pearson correlation coefficients between core therapeutic targets and the relative proportions of the 22 immune cell subtypes were calculated, and the results were visualized using the ggplot2 package.

### 2.10 scRNA-seq analysis

Single-cell RNA sequencing data of cervical cancer (GSE208653), comprising three samples, were obtained from the GEO database. A Seurat object was constructed based on the raw count matrix (Hao et al., 2021), and preliminary filtering of genes and cells was performed: each gene had to be detected in at least three cells, and each cell had to have at least 200 detected genes. To ensure data quality, cells with fewer than 200 or more than 5,000 detected genes were removed, as well as cells in which mitochondrial or ribosomal genes accounted for more than 20% of total expression; this threshold was set based on previous single-cell studies to exclude low-quality or stressed cells. The data were normalized using the LogNormalize method, and 1,500 highly variable genes were identified using FindVariableFeatures. The highly variable genes were then scaled using ScaleData, followed by principal component analysis (PCA). To reduce batch effects among samples, the PCA results were integrated using the Harmony algorithm (Korsunsky et al., 2019). An adjacency graph was constructed based on the integrated features, and clustering was performed using the Louvain algorithm, while t-SNE embeddings were computed for visualization. Cell type annotation was conducted at the cluster level using SingleR, and the predicted labels were mapped back to the identities (Idents) of the Seurat object (Aran et al., 2019). The distribution of pharmacological key targets across different cell subtypes was calculated and visualized using the R package irGSEA. In downstream visualization analyses, up to 200 cells were randomly sampled from each cell type, and gene expression values were Z-score normalized prior to heatmap construction. Bubble plots were used to display the proportion of cells expressing marker genes and their average expression levels across cell types, while feature plots illustrated the distribution of marker genes in the t-SNE space.

### 2.11 Molecular docking and molecular dynamics simulation

The two-dimensional structure of Ori was retrieved from PubChem, and its three-dimensional structure was constructed using ChemOffice and saved in MOL2 format. Proteins and ligands were preprocessed using AutoDock Tools by adding hydrogens and assigning rotatable bonds, and the docking grid box was centered on the protein active pocket. Molecular docking was performed using AutoDock Vina 1.5.6 to screen for the conformation with the lowest binding energy (Eberhardt et al., 2021), and intermolecular interactions were visualized using Discovery Studio 2019 and PyMOL. Molecular dynamics (MD) simulations were conducted using GROMACS 2022 (Abraham et al., 2015), with the AMBER14SB force field applied to proteins and the GAFF2 force field applied to ligands, and partial charges assigned using the RESP method (Zhang et al., 2022). The system was solvated using the TIP3P water model, and Na^+^ and Cl^−^ ions were added to maintain electrostatic neutrality at an ionic concentration of 0.15 M (Nguyen et al., 2014). Long-range electrostatic interactions were treated using the particle mesh Ewald (PME) method, and hydrogen bond constraints were handled using the LINCS algorithm (Mark and Nilsson, 2002). After energy minimization, a 100 ns simulation was performed under the NPT ensemble at 310 K and 1 bar with a time step of 2 fs. Trajectory analyses were carried out using gmx rmsd, gmx rmsf, gmx gyrate, and gmx sasa to evaluate the stability and conformational dynamics of the protein–ligand complex.

### 2.12 Cell culture

SiHa and HeLa cells were cultured in MEM supplemented with 10% fetal bovine serum and 1% penicillin–streptomycin at 37 °C in a humidified atmosphere containing 5% CO_2_. The culture medium was replaced every 2–3 days according to cell growth status, and cells were passaged and used for subsequent experiments when confluence reached approximately 80%–90%.

### 2.13 Cell proliferation assays

Cell proliferative capacity was evaluated using Cell Counting Kit-8 (CCK-8), EdU incorporation, and colony formation assays.

CCK-8 assay: SiHa and HeLa cells were digested with trypsin and seeded into 96-well plates at a density of 5 × 10^3^ cells per well, cultured for 24 h, and then treated with different concentrations of Ori, with a blank control included. After 24 h of treatment, 10 μL of CCK-8 solution was added to each well and incubated for 1 h at 37 °C in the dark, and optical density (OD) was measured at 450 nm using a microplate reader to assess cell proliferation and calculate IC_50_ values.

EdU assay: HeLa cells were seeded at a density of 5 × 10^3^ cells per well in appropriate culture plates, cultured for 24 h, treated with different concentrations of Ori, and simultaneously incubated with EdU (10 μM) for 2 h. After treatment, cells were fixed with 4% paraformaldehyde for 15 min and stained, and the proportion of EdU-positive cells was quantified using ImageJ software to evaluate proliferative activity.

Colony formation assay: HeLa cells in the logarithmic growth phase were seeded into six-well plates at a density of 1 × 10^3^ cells per well, cultured for 24 h, treated with different concentrations of Ori, and maintained in MEM containing 10% FBS for 14 days. At the end of incubation, cells were washed with PBS, fixed with 4% paraformaldehyde for 15 min, stained with 1% crystal violet for 15 min, and the number of colonies per well was quantified using ImageJ.

### 2.14 Cell migration and invasion assay

The Transwell chamber method was used to evaluate the migration and invasion ability of HeLa cells. Migration experiments used uncoated chambers, while invasion experiments involved precoating the upper chamber with Matrigel. HeLa cells treated with different concentrations of Ori (1 × 10^5^ cells/well) were added to serum-free medium in the upper chamber, while medium containing 10% fetal bovine serum was placed in the lower chamber to induce cell migration or invasion. After 24 h of incubation, the cells that had passed through the membrane were washed, stained, and observed under an optical microscope for imaging.

### 2.15 Cell apoptosis detection

HeLa cells were seeded at a density of 5 × 10^5^ cells/well and treated with complete medium containing different concentrations of Ori for 24 h. After treatment, the cells were collected, washed with PBS, and resuspended in 500 μL of 1× Binding Buffer. The cells were transferred to flow cytometry tubes and sequentially incubated with 5 μL of Annexin V-APC and 10 μL of 7-AAD dye. A single dye compensation control group was set. After gentle mixing, the cells were incubated in the dark at room temperature for 5 min and then analyzed by flow cytometry.

### 2.16 Western blot

After HeLa cells were treated with Ori, they were washed twice with pre-chilled PBS, and 100 μL of RIPA lysis buffer containing protease and phosphatase inhibitors was added to each well, followed by lysis on ice for 30 min. The samples were centrifuged at 4 °C, 15,000 rpm for 15 min, and the supernatant was collected. The protein concentration was measured using the BCA method, and the amount of protein loaded was standardized. 20 μg of protein from each well was mixed with 5× sample buffer and denatured by boiling at 100 °C for 10 min. After separation by 10% SDS-PAGE, the proteins were transferred to a PVDF membrane by wet transfer. The membrane was blocked with 5% non-fat milk at room temperature for 1 h, followed by incubation with primary antibodies (GAPDH, BAX, BCL-2, Caspase-3, Cleaved Caspase-3, AKT, p-AKT, PI3K, p-PI3K, E-Cadherin, N-Cadherin, Vimentin, and Snail) at 4 °C overnight. After three washes with TBST, the membrane was incubated with HRP-conjugated secondary antibody at room temperature for 1 h. ECL chemiluminescence was used for detection, and grayscale values were analyzed using ImageJ. The target proteins were normalized to GAPDH, and the phosphorylation levels were expressed as the ratio of p-AKT/AKT and p-PI3K/PI3K.

### 2.17 Statistical analysis

Statistical analysis was performed using GraphPad Prism 10.6.0. All quantitative data were obtained from three independent biological replicates *n* = 3 and are presented as the mean ± standard deviation (SD). Normality and homogeneity of variances were assessed using the Shapiro-Wilk and Levene’s tests, respectively, before applying parametric tests. Statistical comparisons between multiple groups were performed using one-way ANOVA followed by Tukey’s post-hoc test. A p-value of <0.05 was considered statistically significant.

## 3 Results

### 3.1 Predicted targets of Ori

**FIGURE 1:**
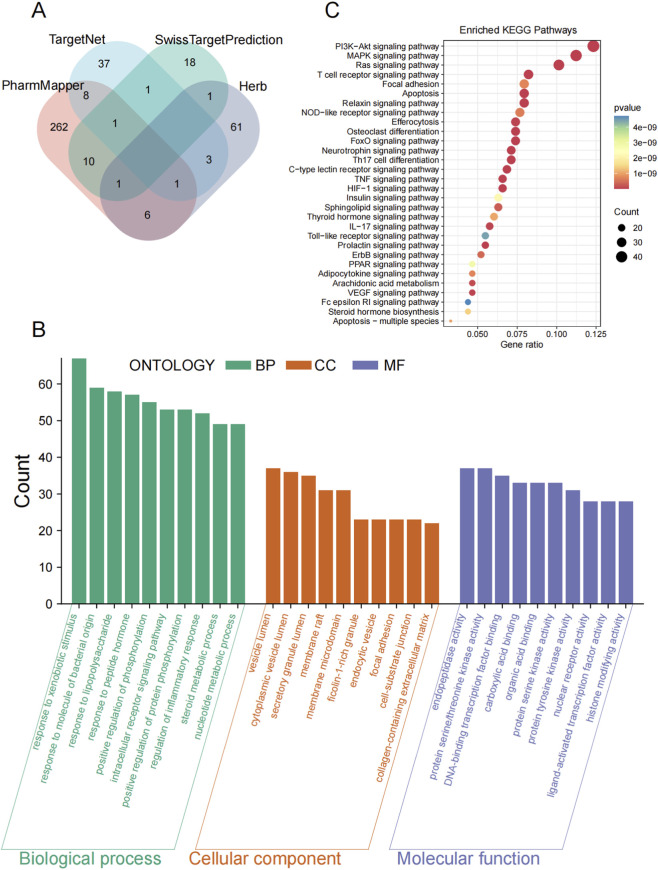
Identification of potential pharmacological targets of oridonin. **(A)** Venn diagram of oridonin potential targets predicted based on four databases. **(B)** Gene Ontology (GO) and **(C)** KEGG pathway enrichment analyses of the predicted targets. These *in silico* analyses provide a predictive landscape of the biological processes and signaling pathways theoretically modulated by oridonin, serving as a basis for further screening.

To explore the multi-target mechanisms of Ori against cervical cancer, we first established its putative target profile. By integrating the SwissTargetPrediction, PharmMapper, TargetNet, and HERB databases, a total of 410 non-redundant candidate targets of Ori were identified (Figure 1A, Supplementary Material 2). The GO enrichment analysis showed that at the biological process (BP) level, these targets are primarily involved in the response to exogenous stimuli, regulation of protein phosphorylation, and G protein-coupled receptor signaling pathways; At the cellular component (CC) level, the targets are mainly distributed in membrane rafts, membrane microdomains, and vesicular compartments; At the molecular function (MF) level, they are significantly enriched in amide binding and protein tyrosine kinase activity (Figure 1B). KEGG pathway analysis revealed that Ori targets are significantly enriched in the PI3K-Akt, MAPK, and Ras signaling pathways, and also show significant enrichment in T cell receptor signaling, apoptosis, and neuroactive ligand–receptor interaction pathways (Figure 1C).

### 3.2 Differential gene expression analysis

**FIGURE 2:**
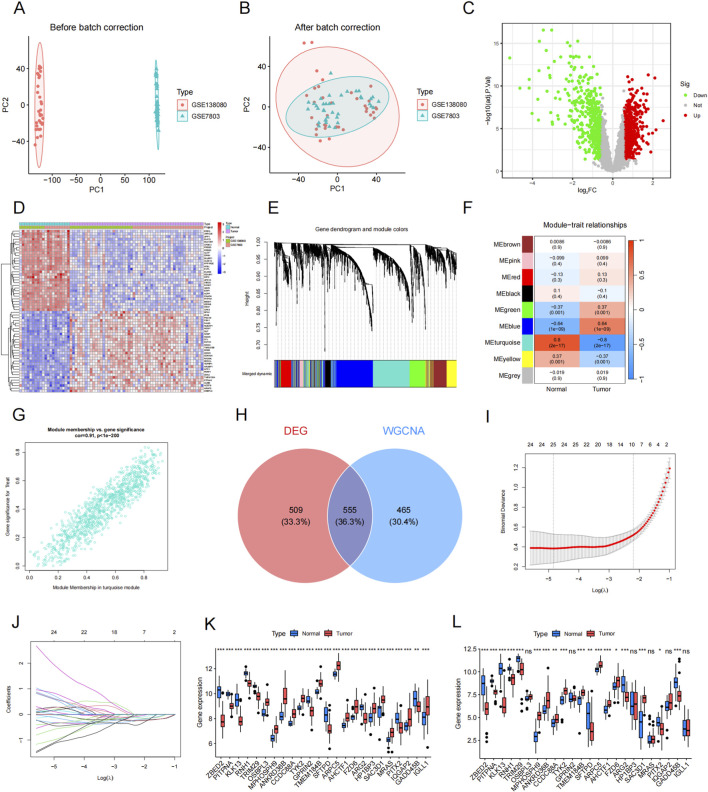
Multi-omic screening of key targets in cervical cancer. **(A)** PCA analysis of samples before batch correction and **(B)** after batch correction, demonstrating the effective elimination of batch effects for reliable transcriptomic profiling. **(C)** Volcano plot identifying computationally predicted differentially expressed genes (DEGs) between tumor and control groups. **(D)** Heatmap illustrating the distinct expression patterns of the top 60 DEGs. **(E)** Gene clustering dendrogram based on dissimilarity measures. **(F)** Correlation analysis between gene co-expression modules and clinical traits, identifying the turquoise module as the most clinically relevant cluster via WGCNA. **(G)** Correlation showing the relationship between feature genes of the turquoise module and module membership. **(H)** Venn diagram showing the intersection between DEGs and turquoise module genes to predict candidate targets. **(I)** LASSO regression (least absolute shrinkage and selection operator model). **(J)** Feature genes selected based on the minimum λ value. **(K)** Expression comparison of identified genes in the training set and **(L)** in the independent validation dataset GSE9750, confirming their consistent differential expression *in silico*.

To determine which of these predicted pharmacological targets are actually relevant to cervical cancer, we first needed to profile the core transcriptomic alterations driven by the disease. After integrating GEO transcriptomic data, batch effects between datasets were markedly reduced. Principal component analysis (PCA) showed that samples clustered primarily according to biological groups, with clear separation between tumor and non-tumor tissues, indicating good comparability of the integrated data (Figures 2A,B). A total of 1,064 differentially expressed genes (DEGs) were identified, including 537 upregulated and 527 downregulated genes (Supplementary Material 3). Volcano plots and heatmaps of DEGs show a balanced distribution of differential genes and effectively distinguished tumor from non-tumor samples (Figures 2C,D).

### 3.3 WGCNA and key gene selection

To further narrow down these transcriptomic alterations to the most critical gene modules driving CESC progression, we applied weighted gene co-expression network analysis (WGCNA). The optimal soft-thresholding power (β) was first determined to ensure that the network conformed to a scale-free topology. After evaluating powers from 1 to 20, β = 5 achieved an R^2^ ≥ 0.8 (Supplementary Figure S2). Based on this parameter, a topological overlap matrix (TOM) was constructed, and nine modules were identified using dynamic tree cutting and average hierarchical clustering, with each module visualized by color coding (Figure 2E). Module–trait correlation analysis revealed varying degrees of association between modules and CESC clinical traits, with the turquoise module showing the strongest correlation with tumorigenesis (cor = −0.8, *p* = 2e-17), and was thus regarded as the hub module (Figure 2F). This module contained 1,221 genes closely related to cervical cancer (cor = 0.91, *p* = 1e-200) (Figure 2G). By integrating differentially expressed genes with hub module genes (removing duplicates), a total of 555 CESC-related genes were obtained (Figure 2H). LASSO regression analysis of 485 intersecting genes identified 24 key genes (Figures 2I,J). These 24 genes exhibited significant expression differences between the control and CESC groups (Figure 2K), and most also showed significant differences in the validation dataset GSE9750 (Figure 2L). Finally, these 24 key genes were merged with 987 known CESC-related genes from the GeneCards database, and after removing duplicates, a final set of 1,009 CESC-related genes was obtained.

### 3.4 Ori anti-CESC targets and PPI network analysis

**FIGURE 3:**
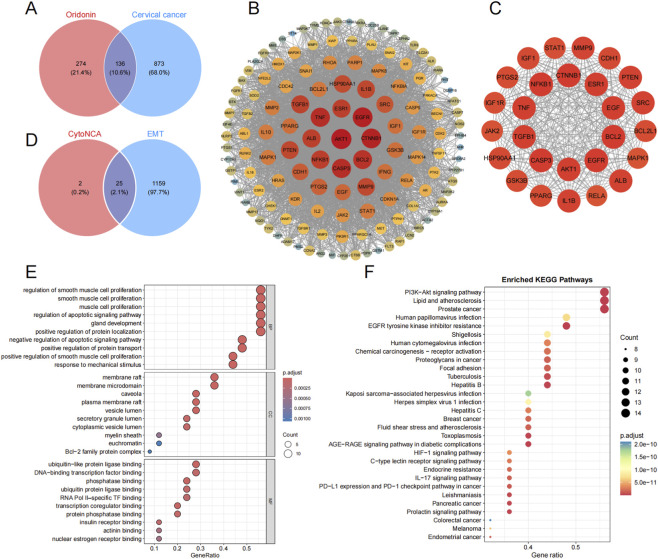
PPI Network Analysis and functional prediction of core targets. **(A)** Venn diagram identifying the predictive targets of Ori for treating CESC. **(B)** PPI network visualizing the theoretical interactions among Ori anti-CESC targets. **(C)** Core gene network computationally extracted using the CytoNCA algorithm to identify topologically significant hub proteins. **(D)** Venn diagram showing the intersection between core genes and EMT-related genes. **(E)** GO enrichment analysis and **(F)** KEGG pathway enrichment analysis predicting the specific biological roles of these intersecting genes in modulating the EMT process.

Having mapped both the predicted Ori targets and the CESC-related genes, we intersected these datasets to explore the potential protein network associated with the drug’s effects. By comparing 1,009 disease-related genes with 410 predicted oridonin (Ori) targets, 136 overlapping targets were identified as potential therapeutic targets of Ori in cervical cancer (Figure 3A). A protein–protein interaction (PPI) network was constructed based on the STRING database, comprising 136 nodes and 3,196 edges, with an average node degree of 46.99 and an average local clustering coefficient of 0.699; PPI enrichment *p* < 1.0e-16, indicating significant interactions among these targets. The PPI network was visualized using Cytoscape, and core genes were screened by integrating multiple topological parameters with the CytoNCA plugin, ultimately identifying highlighting 27 predicted key targets key targets, including AKT1, CASP3, EGFR, BCL2, and ESR1 (Figures 3B,C).

### 3.5 Hub genes and functional enrichment analysis

Recognizing the prominence of migration-related pathways in our initial enrichment, we specifically investigated whether the core targets regulate epithelial-mesenchymal transition. A total of 1,184 EMT-related genes (Supplementary Material 4) were obtained from the dbEMT 2.0 database, and comparison with the 27 core genes identified by CytoNCA yielded 25 overlapping genes, which were considered potential hub genes through which Ori regulates EMT (Figure 3D). GO enrichment analysis indicated that these genes were involved in biological processes (BP) such as smooth muscle cell proliferation, apoptosis, angiogenesis, as well as cell migration and invasion; in cellular components (CC), they were localized to the plasma membrane and cell membrane, vesicle lumen, and intracellular membrane-bound organelles; in molecular functions (MF), they were associated with protein binding, transcription factor binding, and kinase activity (Figure 3E). KEGG pathway analysis revealed that these genes participated in PI3K-Akt, MAPK, and TNF signaling pathways, as well as pathways related to infection and metabolism (Figure 3F). These results suggest that these hub genes may regulate EMT and cervical cancer progression through multiple signaling pathways, providing potential targets for Ori’s therapeutic effects.

### 3.6 Immune infiltration analysis

**FIGURE 4:**
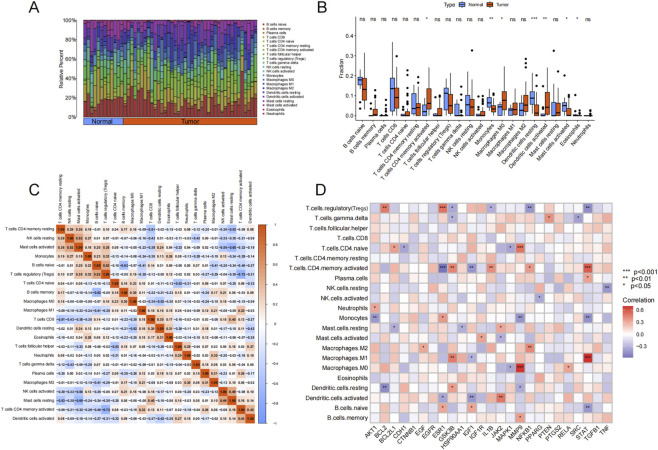
Immune infiltration analysis and target correlation. **(A)** Heatmap and **(B)** boxplot showing the distribution of 22 immune cell types computationally inferred from the GSE7803 and GSE13080 datasets, respectively. **(C)** Correlation matrix illustrating the predicted relationships among the 22 immune cell types. **(D)** Correlation analysis evaluating the theoretical association between 25 key EMT targets and immune cell abundance, predicting potential immunomodulatory effects.

Beyond direct tumor cell targeting, therapeutic efficacy often depends on the tumor immune microenvironment. We therefore explored the association between Ori’s core targets and immune cell infiltration. Immune infiltration analysis of the GSE7803 and GSE13080 datasets was performed using the CIBERSORT algorithm and the LM22 signature gene matrix to assess differences in the inferred immune microenvironment between CESC and control groups. The results showed significant differences in the estimated proportions of multiple immune cell types between the two groups (Figures 4A,B), indicating distinct immune expression profiles in CESC. Heatmaps of immune cell abundance revealed complex correlations among these cells (Figure 4C), reflecting the interactive network of the tumor microenvironment in CESC initiation, progression, and metastasis. Pearson correlation analysis indicated that multiple targets were significantly associated with inferred immune cell proportions (*p* < 0.05), with core targets AKT1, STAT1, STAT3, MAPK1, NFKB1, and PTGS2 showing significant correlations with various immune cells, mainly monocytes and macrophage subsets (M0 and M1). These spatial correlations reveal a significant statistical association between the expression of these targets and the enrichment of innate immune components within the tumor microenvironment. Among T cell subtypes, regulatory T cells (Tregs) and CD4^+^ T cells were significantly correlated with multiple core genes (Figure 4D), highlighting that T cell–related immune features dominate the CESC immune microenvironment and suggesting that T cells may be key targets of Ori’s immunoregulatory effects.

### 3.7 Single-cell transcriptome sequencing and hub gene distribution

**FIGURE 5:**
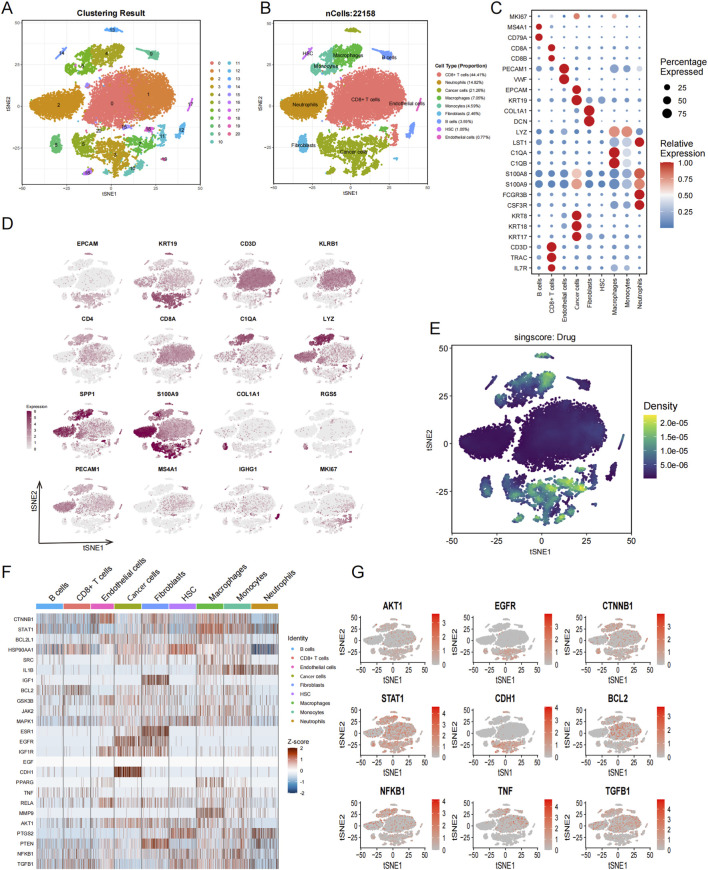
Identification of potential target cell types of Ori based on scRNA-seq data. **(A)** t-SNE visualization of all cells colored according to clustering results. **(B)** Cell type annotation based on classical marker genes, profiling tumor heterogeneity at single-cell resolution **(C)** Dot plot and **(D)** t-SNE plots visualizing the expression distribution of canonical marker genes. **(E)** Computational prediction of the Ori action pathway at the single-cell level. **(F)** Heatmap and **(G)** t-SNE plots revealing the expression of 25 core genes, computationally pinpointing specific cell types theoretically targeted by oridonin.

To resolve these bulk-tissue correlations and pinpoint the precise cellular compartments expressing Ori targets, we integrated single-cell RNA sequencing data. Single-cell RNA sequencing data from three cervical cancer samples were analyzed, and based on Seurat clustering analysis (resolution = 0.6), a total of 20 cell clusters were identified (Figure 5A). A total of 22,158 high-quality cells were obtained and annotated into nine major cell types using classical marker genes: CD8^+^ T cells, neutrophils, tumor cells, macrophages, monocytes, fibroblasts, B cells, hematopoietic stem cells, and vascular endothelial cells (Figure 5B). Marker genes corresponding to each cell type exhibited distinct expression patterns, validating the accuracy of our cell annotations (Figures 5C,D) and reflecting the cellular heterogeneity of cervical cancer tissues. To assess the potential activity of drug target genes across different cell types, irGSEA scoring was applied, revealing that most targets were primarily active in tumor cells and macrophages, suggesting that tumor cells and the immune microenvironment may serve as potential therapeutic targets (Figure 5E). Furthermore, a heatmap illustrated the expression distribution of hub genes across the nine cell clusters (Figure 5F), providing a visual reference for the overall response of each cell type to key therapeutic genes. t-SNE plots (Figure 5G) further displayed the expression patterns of nine key therapeutic genes within the single-cell landscape.

### 3.8 Affinity analysis

**FIGURE 6:**
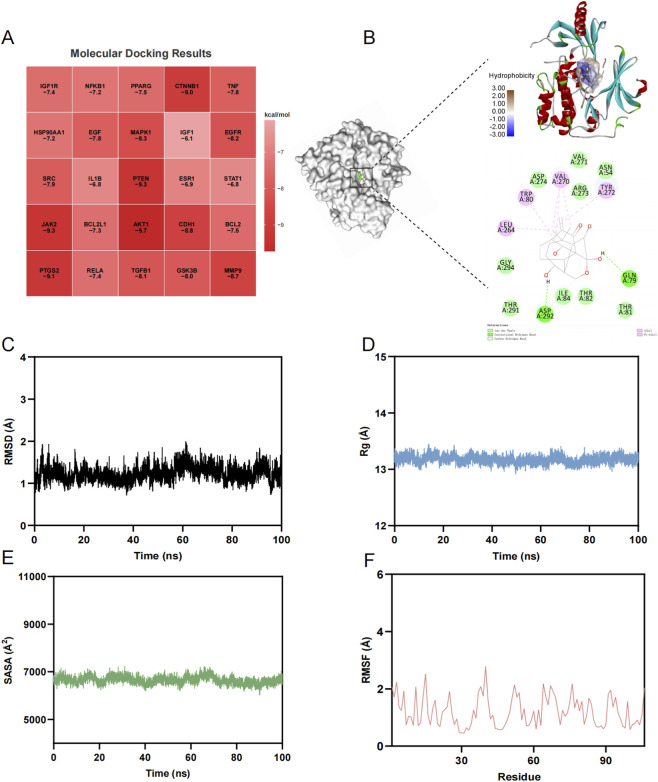
In silico analysis of binding affinity and molecular docking simulation. **(A)** Heatmap of computationally predicted binding affinities between Ori and the 25 core genes. **(B)** Representative binding mode of Ori with the AKT protein predicted by molecular docking, showing key interacting residues. **(C)** Time-dependent RMSD curve **(D)** radius of gyration (Rg), **(E)** solvent-accessible surface area (SASA), and **(F)** root mean square fluctuation (RMSF) theoretically predicting the structural stability and dynamic behavior of the Ori-AKT complex *in silico*.

While the preceding multi-omics analyses underscored the biological significance of the core targets, their capacity to physically interact with Ori required structural evaluation. Molecular docking results showed that Ori exhibited binding energies below −5.0 kcal/mol for all targets, indicating potential binding activity (Figure 6A). Among them, Ori displayed the strongest binding to AKT1, with a binding energy of −9.7 kcal/mol. Docking analysis revealed that Ori deeply embedded into the surface cavity of the AKT1 protein, complementing the target structure and forming a stable binding mode. Interaction analysis indicated that the ligand formed hydrogen bonds with ASP292 and GLN79 residues, hydrophobic interactions with LEU264, TRP80, VAL270, and TYR272 residues, and van der Waals interactions with GLY294, THR291, ILE84, THR81, ARG273, and ASP274 residues, collectively maintaining the structural stability of the complex (Figure 6B). The stability of the Ori–AKT1 complex was further evaluated via a 100 ns molecular dynamics simulation. RMSD analysis showed that the complex reached equilibrium at approximately 60 ns, after which RMSD values fluctuated around 1.4 Å, indicating good structural stability during the simulation. The radius of gyration (Rg) remained relatively constant throughout the simulation, suggesting that the overall protein conformation remained tightly packed without significant changes. Solvent-accessible surface area (SASA) analysis showed minimal fluctuations, indicating that ligand binding did not significantly affect the overall protein structure. RMSF analysis revealed that most residues fluctuated less than 2 Å, suggesting low flexibility of the complex and overall conformational stability (Figures 6C–F). However, it is important to note that while molecular docking and MD simulations indicate favorable binding thermodynamics, these approaches serve as predictive computational models. The direct physical engagement and specific inhibition of AKT1 by Ori in living cells remain to be experimentally established through future biochemical assays, such as direct kinase activity measurements or target knockdown studies.

### 3.9 Ori inhibits cervical cancer cell proliferation, migration, and invasion and induces apoptosis

**FIGURE 7:**
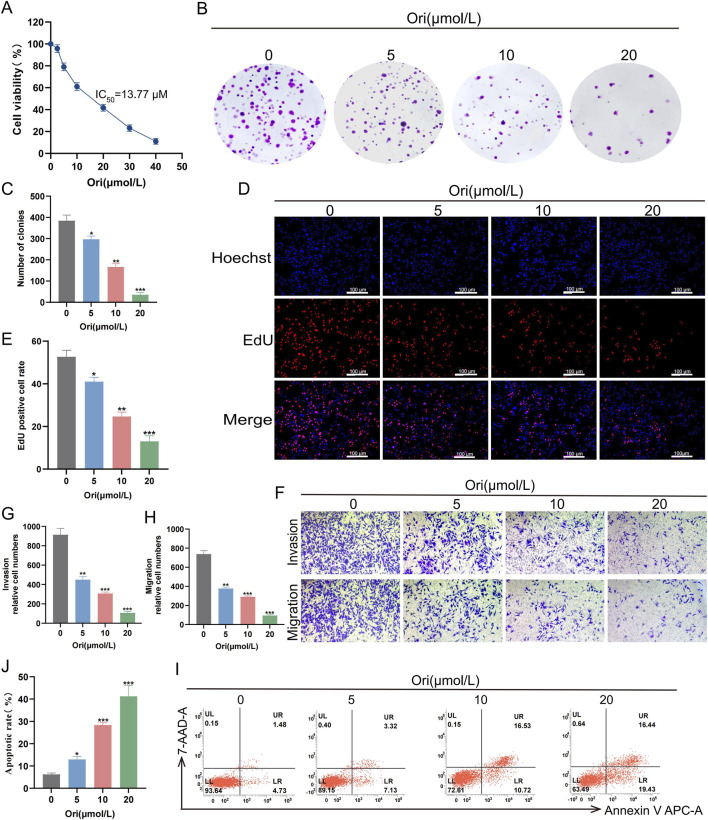
Experimental validation (*in vitro*): Inhibitory effects of Ori on HeLa cells. **(A)** Cell viability measured by CCK-8 assay, experimentally validating the dose-dependent cytotoxicity of Ori. **(B)** Representative images and **(C)** Quantitative analysis of colony formation, confirming sustained anti-proliferative effects *in vitro*. **(D)** EdU staining images and **(E)** statistical analysis of EdU-positive cells, biologically demonstrating the inhibition of DNA synthesis. **(F–H)** Transwell migration and invasion assays, demonstrating the suppression of metastatic potential. **(I)** Flow cytometric plots and **(J)** statistical analysis of apoptosis rates, providing *in vitro* confirmation of Ori-induced apoptosis. Data are presented as the mean ± SD from *n* = 3 independent biological replicates. Statistical significance was determined using one-way ANOVA followed by Tukey’s post-hoc test. Scale bar = 100 μm *p < 0.05, **p < 0.01, ***p < 0.001.

The comprehensive *in silico* analyses generated a clear hypothesis: Ori exerts anti-CESC effects by targeting the PI3K/AKT pathway and modulating EMT. To empirically test this, we evaluated Ori’s direct pharmacological impact on CESC cells *in vitro*. To investigate the effect of Ori on the viability of cervical cancer cells, CCK-8 assays were performed according to the manufacturer’s instructions. The results showed that the viability of HeLa cells gradually decreased with increasing concentrations of Ori (Figure 7A). The exact IC_50_ value was calculated at 13.77 μM. This finding demonstrates that Ori effectively inhibits the proliferation of cervical cancer cells. In addition, colony formation assays and EdU staining in HeLa cells further supported this conclusion (Figures 7B–E). Transwell assays were used to evaluate the effects of Ori on the migration and invasion of HeLa cells. The results demonstrated that Ori treatment significantly reduced the number of HeLa cells, regardless of whether the upper chamber was coated with Matrigel, and exerted a pronounced inhibitory effect at 20 μM (Figures 7F–H), indicating that Ori effectively blocks cell migration and invasion. Subsequently, the effect of Ori on apoptosis in HeLa cells was assessed by flow cytometry. After 24 h of treatment with different concentrations of Ori, HeLa cells were stained with Annexin V–7AAD/APC. The results showed that Ori treatment significantly increased the proportion of apoptotic cells in a dose-dependent manner (Figures 7I,J), indicating that Ori suppresses cell growth by promoting apoptosis. Importantly, to confirm that these anti-tumor phenotypes are not limited to a single cell model, parallel functional assessments were conducted in the SiHa cell line. Consistent with the observations in HeLa cells, Ori treatment exhibited similar dose-dependent inhibitory effects on SiHa cell proliferation, migration, and invasion, along with the induction of apoptosis (Supplementary Figure S3), indicating that these phenotypic responses are broadly conserved across different CESC backgrounds.

### 3.10 Regulation of apoptosis, the PI3K/AKT signaling pathway, and EMT-related proteins by Ori

**FIGURE 8:**
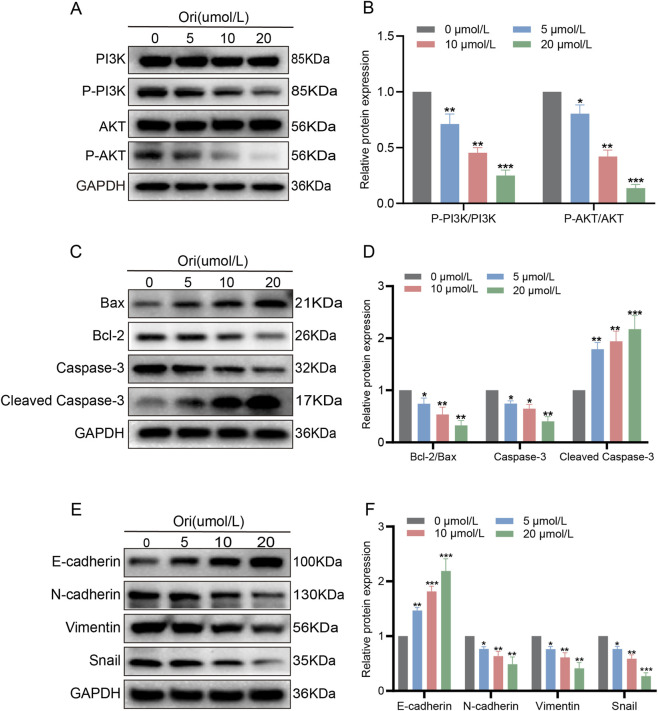
Experimental validation (*in vitro*): Regulatory effects of Ori on PI3K/AKT and EMT markers. **(A)** Representative Western blot images and **(B)** quantitative analysis of PI3K, p-PI3K, AKT, and p-AKT, experimentally validating the protein-level inhibition of this signaling cascade. **(C)** Western blot results and **(D)** quantitative analysis of apoptosis-related proteins (BAX, BCL-2, caspase-3, and cleaved caspase-3), mechanistically verifying the activation of mitochondrial apoptosis. **(E)** Representative Western blot images and **(F)** quantitative analysis of EMT markers (E-cadherin, N-cadherin, vimentin, and Snail), providing definitive biological proof that Ori reverses the EMT phenotype, consistent with the bioinformatic predictions. Data are presented as the mean ± SD from *n* = 3 independent biological replicates. Statistical significance was determined using one-way ANOVA followed by Tukey’s post-hoc test. *p < 0.05, **p < 0.01, ***p < 0.001.

Having confirmed Ori’s robust suppression of cancer cell phenotypes, we finally verified whether these effects were mechanistically driven by the predicted PI3K/AKT and EMT pathways.Western blot analysis showed that, with increasing concentrations of Ori, the pro-apoptotic protein BAX was upregulated in a dose-dependent manner, whereas the anti-apoptotic protein BCL-2 was downregulated. Concurrently, the level of total caspase-3 decreased while active cleaved caspase-3 increased. These combined protein alterations indicate that Ori induces the intrinsic mitochondrial apoptotic pathway in HeLa cells by ultimately promoting executioner caspase-3 activation (Figures 8A,B). Analysis of the PI3K/AKT signaling pathway demonstrated that the phosphorylated forms p-PI3K and p-AKT decreased significantly with increasing Ori concentration, while total PI3K and total AKT expression remained relatively stable, indicating that Ori primarily inhibits activation of this pathway (Figures 8C,D). Analysis of EMT-related proteins showed that E-cadherin expression was upregulated, whereas N-cadherin, vimentin, and Snail were downregulated, suggesting that Ori may suppress epithelial–mesenchymal transition (Figures 8E,F). Furthermore, to validate the broad applicability of these mechanistic pathways across different cervical cancer backgrounds, we performed parallel Western blot analyses in the SiHa cell line. Consistent with the findings in HeLa cells, Ori treatment in SiHa cells exhibited similar dose-dependent regulatory effects on apoptosis, PI3K/AKT signaling inhibition, and EMT suppression (Supplementary Figure S4). These overall results are consistent with predictions from single-cell analysis and irGSEA scoring, further supporting a potential mechanism by which Ori exerts anti-cervical cancer effects through induction of apoptosis, inhibition of the PI3K/AKT signaling pathway, and interference with the EMT process.

## 4 Discussion

Cervical cancer remains a major malignancy among women worldwide, with particularly high incidence and mortality rates in some developing countries (Hamid et al., 2025). Although the implementation of screening strategies and the application of HPV vaccines have improved disease prevention and control to some extent, the clinical benefits of current therapeutic regimens remain limited for patients with advanced or recurrent CESC (Zhang et al., 2025). Surgery, radiotherapy, and platinum-based chemotherapy remain the main treatment modalities; however, drug resistance, recurrence, and treatment-related adverse effects continue to constrain further improvements in therapeutic efficacy (Deng et al., 2022; Liu et al., 2022; Tewari et al., 2024). The emergence of resistance mutations further complicates treatment, highlighting the critical need to explore the comparative mechanistic interpretation of how mutational landscapes impact drug targets and resistance mechanisms (Bano and Raza, 2025). Therefore, continued efforts to identify novel candidate drugs and to elucidate their potential mechanisms of action remain of substantial clinical relevance.

Ori is a bioactive diterpenoid compound derived from traditional Chinese medicine (Li et al., 2016) and has been reported to exhibit antitumor activity in multiple tumor models, with mechanisms involving inhibition of cell proliferation, induction of apoptosis, regulation of epithelial–mesenchymal transition (EMT), and modulation of tumor energy metabolism (Liu et al., 2024; Guo et al., 2025; Ma et al., 2025). However, the molecular mechanisms underlying the effects of Ori in cervical cancer remain incompletely understood, particularly with respect to EMT-related regulation and its potential association with the tumor immune microenvironment. In this context, the present study integrated network pharmacology, transcriptomic analysis, single-cell RNA sequencing, and *in vitro* experiments to explore the multi-target antitumor effects of Ori in CESC. The integration of computational multi-omics with machine learning and network pharmacology has increasingly been recognized as a powerful strategy for systematically identifying multi-target drug mechanisms in complex biological systems, such as antimicrobial resistance and oncology (Bano et al., 2025). Crucially, rather than merely reiterating the well-documented suppressive effects of Ori on the PI3K/AKT pathway and EMT observed in conventional bulk-tumor models, our study critically advances the field by layering scRNA-seq to map these predicted targets to specific cellular compartments. This high-resolution and cell-type-specific mapping provides novel mechanistic insights that were previously unattainable using bulk transcriptomics. By demonstrating that Ori-associated targets are highly enriched in specific microenvironmental niches, particularly macrophages, this study contributes new knowledge: Ori exerts its systemic anti-CESC effects not only through direct cytotoxicity on epithelial tumor cells but also by dynamically modulating the innate immune microenvironment.

Using multiple target prediction databases, a total of 410 potential targets of Ori were identified, and functional enrichment analysis indicated that these targets were mainly enriched in classical cancer-related signaling pathways, including PI3K–Akt, MAPK, and Ras, as well as biological processes related to protein phosphorylation regulation, G protein–coupled receptor signaling, and membrane-associated molecular functions (Li et al., 2024; Hou et al., 2025; Zhao et al., 2025). These enrichment patterns suggest that Ori-associated targets are broadly involved in signaling pathways relevant to tumor cell growth, survival, and migratory behavior (Chen and Shen, 2024). Furthermore, by integrating GeneCards-derived cervical cancer–related genes with GEO transcriptomic data, 1,009 CESC-associated genes were obtained, and intersection analysis yielded 136 candidate targets potentially relevant to the anti-CESC activity of Ori. Subsequent topological analysis identified 27 core targets, among which 25 were closely associated with EMT-related processes. Crucially, rather than viewing EMT merely as an isolated phenotypic consequence, our topological findings advance the field by illustrating how Ori concurrently modulates multiple interlinked EMT-associated nodes within a systemic pharmacological network. Molecular docking analysis further indicated favorable binding potential between Ori and these 25 proteins, and molecular dynamics simulations of the Ori–AKT1 complex provided supportive structural insights into the stability of this interaction. However, it is essential to note that these computational models are strictly hypothesis-generating. While they suggest potential relevance in the context of Ori’s antitumor activity, the definitive direct engagement of these targets requires rigorous future biochemical and biophysical validation.

Immune infiltration analysis further suggested that Ori-related targets may be associated with the tumor immune microenvironment. In cervical cancer tissues, multiple immune cell subsets showed significant correlations with the identified core targets, with monocytes and macrophage subsets exhibiting particularly prominent associations. It is important to note that these CIBERSORT-derived correlations are indirect; while they imply that Ori-associated targets are highly expressed in microenvironments rich in innate immune cells, they do not establish a direct causal immunomodulatory effect. Regulatory T cells and CD4^+^ T cells also displayed certain correlations, suggesting these targets may also be spatially or contextually relevant in adaptive immune microenvironments, rather than indicating a direct regulatory role. To further characterize the cellular distribution of core targets, single-cell RNA sequencing data from three cervical cancer cases were analyzed. Nine major cell types were identified, including tumor cells, macrophages, CD8^+^ T cells, monocytes, B cells, fibroblasts, vascular endothelial cells, hematopoietic stem cells, and neutrophils. Analysis of target gene activity indicated that core targets were predominantly enriched in tumor cells and macrophages, providing cell-type–specific insights into the potential biological contexts in which Ori-associated targets may function. However, given the limited sample size (*n* = 3),these cell-type-specific findings must be interpreted cautiously as strictly hypothesis-generating. Larger clinical cohorts are required to adequately account for inter-sample variability and to confirm the generalizability of these target distributions across broader patient populations. While these observations are consistent with the immune-regulatory associations of Ori reported in other tumor types (Hwang and Chang, 2023; Chen and Shen, 2024), the restricted sample size and *in silico* nature of this analysis mean they primarily suggest potential multi-target interactions rather than providing definitive proof of systemic antitumor characteristics, thereby warranting rigorous future *in vivo* and functional validation.

*In vitro* functional experiments provided experimental support for the antitumor activity of Ori in cervical cancer cells. CCK-8 assay results showed that Ori significantly reduced the viability of HeLa and SiHa cells in a dose-dependent manner, with half-maximal inhibitory concentrations (IC50) of 13.77 μM and 14.23 μM, respectively. EdU incorporation and colony formation assays further demonstrated a marked suppression of HeLa cell proliferation following Ori treatment. Flow cytometry analysis revealed a significant increase in apoptotic cell populations. Importantly, to address the inherent histological and viral heterogeneity of cervical cancer, our experimental design deliberately incorporated both the HeLa and SiHa cell lines. HeLa serves as a representative model for HPV-18 positive cervical adenocarcinoma, while SiHa models HPV-16 positive cervical squamous cell carcinoma. Our parallel functional and Western blot analyses confirmed that the pharmacological mechanisms of oridonin are highly conserved across these two most prevalent clinical subtypes. In both HeLa and SiHa cells, Ori treatment led to the upregulation of pro-apoptotic proteins BAX and cleaved caspase-3, accompanied by downregulation of the anti-apoptotic protein BCL-2, indicating activation of the intrinsic mitochondrial apoptotic pathway and its downstream executioner cascade. In addition, Ori treatment significantly reduced the phosphorylation levels of key proteins in the PI3K/AKT signaling pathway in both cell lines, while total protein expression remained relatively stable, suggesting an effect primarily on pathway activation status. Analysis of EMT-related markers in both HeLa and SiHa cells showed increased expression of E-cadherin and decreased expression of N-cadherin, vimentin, and Snail, indicating an association between Ori treatment and EMT-related phenotypic changes. Transwell assays further showed that Ori treatment reduced HeLa cell migration and invasion. Collectively, rather than merely confirming the well-known suppressive effects of Ori on the PI3K/AKT and EMT pathways, these *in vitro* findings critically serve to validate our specific computational network models, successfully grounding our multi-omics hypotheses in empirical biological systems. However, interpreting these *in vitro* concentrations must be done in the context of Ori’s known pharmacokinetic profile. Previous pharmacokinetic studies show that Ori has poor aqueous solubility and low oral bioavailability (typically 4%–11% in rodent models) (Li et al., 2021), making it difficult to achieve and maintain such high micromolar concentrations in the bloodstream (∼14 μM) with conventional formulations. Although these concentrations effectively target the PI3K/AKT and EMT-related pathways *in vitro*, translating these doses to an *in vivo* setting remains a major challenge. Future translational research should assess Ori’s *in vivo* tissue distribution and explore advanced drug delivery systems such as targeted nanocarriers, liposomes, or structural modifications (Li et al., 2021; Li et al., 2024; Ma et al., 2025) to improve its bioavailability and ensure that therapeutic levels can be reached within the tumor microenvironment.

Despite promising results, several methodological limitations warrant consideration. The computational nature of network pharmacology and docking inherently relies on existing databases, which may introduce selection bias and false-positive associations. Alongside these algorithmic constraints, the small scRNA-seq sample size *n* = 3 limits the evaluation of inter-patient heterogeneity and the generalizability of our cell-type-specific findings. Biologically, key targets like AKT1 lack functional validation via gene-editing approaches (e.g., siRNA or CRISPR/Cas9), meaning their precise causal roles in mediating Ori’s effects remain predictive.Additionally, the multi-target nature of natural compounds like oridonin inherently carries the risk of off-target effects and non-specific toxicity in healthy tissues. Comprehensive kinase profiling and rigorous toxicological evaluations are therefore required to fully understand its systemic safety profile. Furthermore, the absence of *in vivo* models leaves a significant translational gap. *In vitro* systems cannot fully replicate the complex tumor microenvironment or systemic pharmacokinetics, making the clinical translation of the relatively high effective concentrations (IC_50_ ∼14 μM) observed here highly challenging. Similarly, CIBERSORT-derived immune infiltration patterns are correlational and do not prove direct immunomodulation. Crucially, alongside novel targeted delivery strategies, future *in vivo* studies utilizing well-designed animal models are strictly essential to validate these computational predictions, evaluate systemic toxicity, and establish the true clinical efficacy and safety profile of oridonin before therapeutic feasibility can be confirmed.

## 5 Conclusion

In conclusion, this study integrated network pharmacology, transcriptomic analysis, single-cell RNA sequencing, and *in vitro* experiments to evaluate the multi-target pharmacological network of oridonin in cervical cancer. Our *in vitro* results demonstrate that oridonin inhibits cell proliferation, induces apoptosis, and suppresses the activation of the PI3K/AKT signaling pathway alongside a reversion of EMT markers toward an epithelial phenotype. Furthermore, our computational analyses, incorporating immune infiltration and single-cell mapping, generated the hypothesis that oridonin-associated targets are predominantly enriched in tumor cells and macrophages, suggesting potential interactions within the broader immune microenvironment. However, because these computational predictions currently lack direct target-engagement validation and *in vivo* confirmation, clinical translational claims remain premature. Ultimately, this integrative study provides cell-type-specific, hypothesis-driven directions for future research, which must prioritize rigorous *in vivo* modeling and direct mechanistic validation to fully elucidate oridonin’s systemic antitumor properties.

## Data Availability

The datasets presented in this study can be found in online repositories. The names of the repository/repositories and accession number(s) can be found in the article Supplementary Material.

## Supplementary material

SUPPLEMENTARY FIGURE Technical roadmap of this study, soft-thresholding power selection in WGCNA, and *in vitro* experimental validation of Ori on SiHa cells.

SUPPLEMENTARY MATERIAL 1 genes with a Relevance Score greater than 20 were selected to construct a disease-related gene set.

SUPPLEMENTARY MATERIAL 2 By integrating the SwissTargetPrediction, PharmMapper, TargetNet, and HERB databases, a total of 410 non-redundant candidate targets of Ori were identified.

SUPPLEMENTARY MATERIAL 3 A total of 1,064 differentially expressed genes (DEGs) were identified, including 537 upregulated and 527 downregulated genes.

SUPPLEMENTARY MATERIAL 4 A total of 1,184 EMT-related genes were obtained from the dbEMT 2.0 database.

SUPPLEMENTARY MATERIAL 5 Original Western Blot Images.
