# Supplementary material for: Integrative network pharmacology, molecular dynamics simulation, and single-cell RNA sequencing strategies reveal the multi-target mechanisms of oridonin against cervical cancer
Source: Front Pharmacol. 2026 Jun 17;17:1788376. doi: 10.3389/fphar.2026.1788376 (PMC13318904; doi:10.3389/fphar.2026.1788376)
Supplement: Supplementary file 1 [file Supplementaryfile5.pdf]

(A)

Supplementary Material Panels (A), (B), (C), (D), (E), (F)present the original Western blot (WB) bands and corresponding quantitative data in HeLa cells. Rawdataofwesternblot (a) andstatisticalanalysis (b) .Raw data of three independent replicates and statistical analysis of Western blot corresponding to A and B in Figure 8

(a)

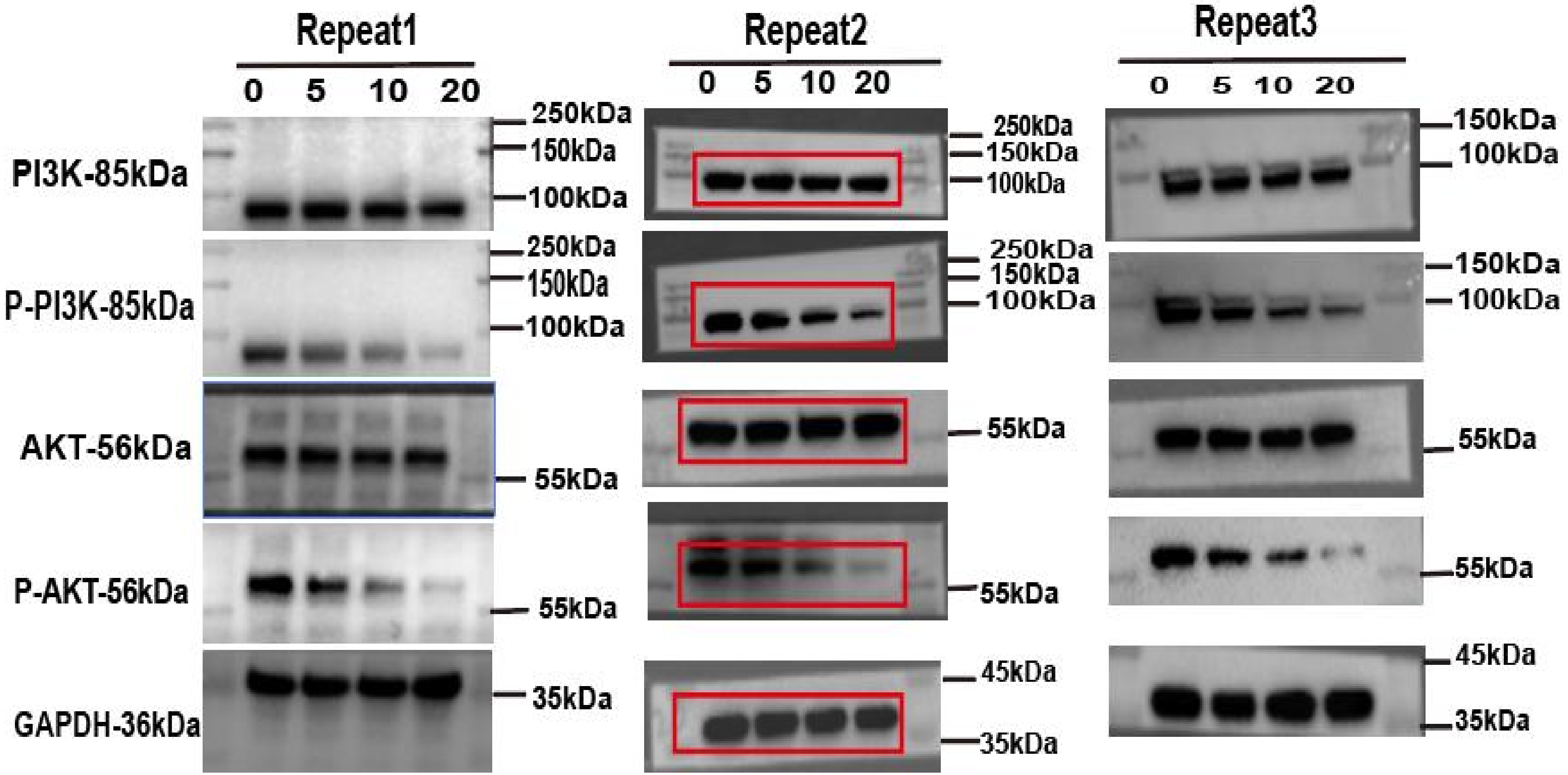

(B)

(b)Statistic alanalysis of data was used ImageJ

Oridonin（μmol/L）	Repeat1				Repeat2				Repeat3			
	0	5	10	20	0	5	10	20	0	5	10	20
PI3K	39821.459	40991.095	31990.01	31919.53	41758	40994.338	40982.439	42199.952	36758.359	36374.338	36382.439	35899.982
P-PI3K	38990.974	24626.068	13150	6169.004	39075	28217.702	19480.010	10100.004	39975.267	31169.223	17241.899	11519.874
P-PI3K/PI3K/Control	1	0.6135623	0.419821	0.197384	1	0.735595789	0.507963894	0.25577086	1	0.787944484	0.435770702	0.2950654
AKT	41077.024	39142.246	38945.15	39998.27	45484	45991.803	45971.599	48999.945	34576.258	33093.966	33619.057	35884.602
P-AKT	39999.602	31311.945	15103.19	5328.326	43735	38834.752	16802.095	5008.752	32772.53	22517.145	15479.999	5801.04
P-AKT/AKT/Control	1	0.8215001	0.398253	0.136802	1	0.878159864	0.380108262	0.10630836	1	0.717848056	0.485795426	0.1705555

Note: **(A)** and **(B)** represent the raw data from three independent Western blot replicates **(a)** and the statistical analysis of the three independent replicates **(b)** corresponding to panels A and B in Figure 8, respectively. The red boxes in panel **(a)** indicate the original Western blot bands used in Figure 8A, and the red-marked values in panel **(b)** are the data used for the statistical graph in Figure 8B.

(C)

Supplementary Rawdataofwesternblot（c）andstatisticalanalysis（d）
Raw data of three independent replicates and statistical analysis of Western blot corresponding toCand D in Figure 8 (c)

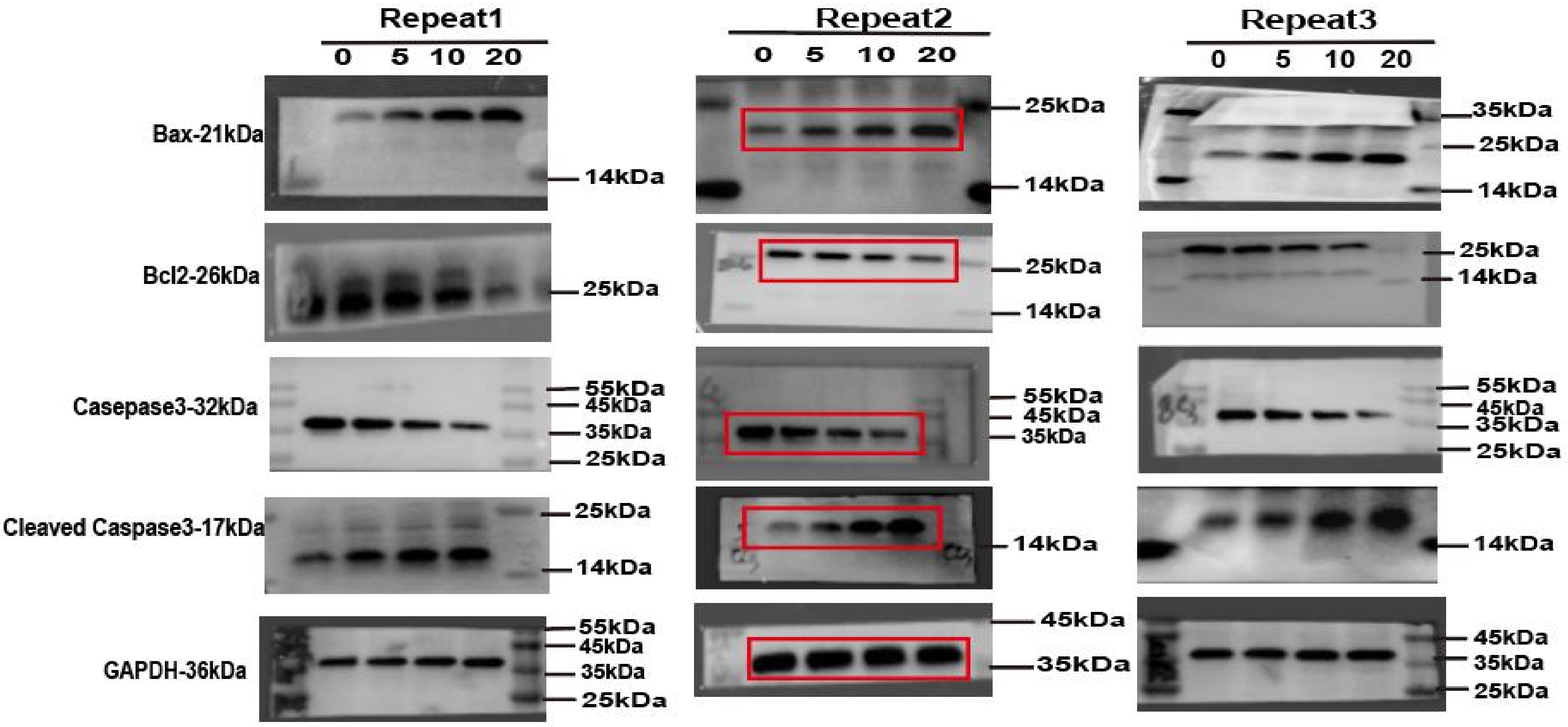

(D)

(d) Statistic alanalysis of data was used ImageJ

Oridonin (μmol/L)	Repeat1				Repeat2				Repeat3			
	0	5	10	20	0	5	10	20	0	5	10	20
Bax	15211.995	18973.359	31269.874	39397.803	29572.945	27577.643	32160.48	36047.572	19362.731	30173.924	32464.995	41304.853
Bcl-2	37146.581	36987.409	29799.56	22327.924	35941.016	30154.179	26245.915	18239.924	43001.501	41283.924	39226.217	30040.823
Bax/Bcl-2/Control	1	0.79832	0.39025731	0.23208318	1	0.899693513	0.67149599	0.41634297	1	0.616073	0.5440566	0.32748676
GAPDH	34478.974	34469.317	34899.931	37909.137	38656.581	36713.823	39155.894	38797.288	33469.439	35451.731	38717.631	41999.088
Caspase3	36212.066	31998.309	25599.288	13889.317	41777.43	32418.48	29221.238	21311.903	41333.187	34894.267	26374.317	18495.924
Caspase3/GAPDH/Control	1	0.88388422	0.69840019	0.34884943	1	0.817042708	0.69053101	0.50827949	1	0.79701441	0.55159711	0.35660359
Cleaved Caspase3	20188.317	34119.946	35295.439	42060.995	20188.317	37019.946	40995.439	44820.095	13939.539	25745.995	33786.652	42300.581
Cleaved Caspase3 /GAPDH//Control	1	1.69055722	1.72722231	1.89491556	1	1.930765347	2.00475692	2.21204893	1	1.74370196	2.09525268	2.4182795

Note: (C)and **(D)** represent the raw data from three independent Western blot replicates **(c)** and the statistical analysis of the three independent replicates **(d)** corresponding to panels C and D in Figure 8, respectively. The red boxes in panel **(c)** indicate the original Western blot bands used in Figure 8C, and the red-marked values in panel **(d)** are the data used for the statistical graph in Figure 8D.

(E) Supplementary Rawdataofwesternblot (e) andstatisticalanalysis (f)

Raw data of three independent replicates and statistical analysis of Western blot corresponding to Eand F in Figure 8

(e)

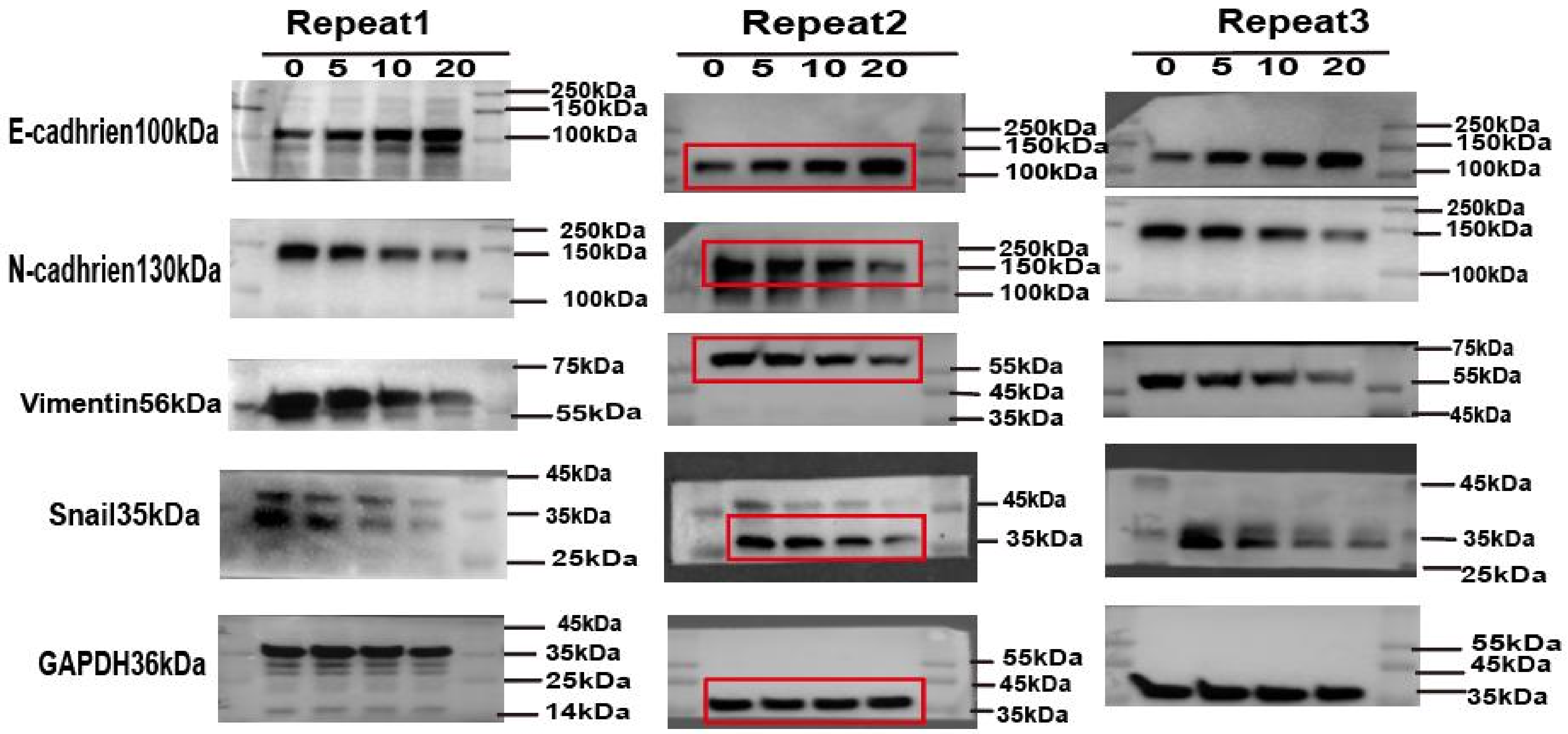

(F)

(f)

Statistic alanalysis of data was used ImageJ

Oridonin (μmol/L)	Repeat1				Repeat2				Repeat3			
	0	5	10	20	0	5	10	20	0	5	10	20
E-cadhrien	25470.874	40299.782	46835.711	55605.539	19451.853	27932.095	34280.853	40213.782	25507.13	34434.439	39935.853	50596.217
N-cadhrien	38600.551	29039.037	20598.681	12911.397	34006.874	27901.723	24801.995	19259.518	38803.8	27597.338	21914.622	18786.095
Vimentin	49026.773	41585.016	28806.602	17299.924	33998.652	25133.43	18872.995	11991.853	36700.63	25182.338	21752.095	16088.66
Snail	49026.272	36385.212	31206.622	13569.369	33998.324	28001.243	17672.295	11701.256	36700.63	25992.338	18952.095	7088.66
GAPDH	43894.208	45702.161	43318.103	43242.169	41974.418	42722.267	43318.853	44342.288	43883.38	40233.945	36654.56	36207.924
E-cadhrien/GAPDH/Control	1	1.5196002	1.86324969	2.2160214	1	1.41082428	1.70764808	1.956953585	1	1.47244457	1.874448508	2.40410251
N-cadhrien/GAPDH/Control	1	0.7225355	0.54073402	0.339531	1	0.80611073	0.70668787	0.536099357	1	0.77571172	0.676132408	0.58675753
Vimentin/GAPDH/Control	1	0.8146556	0.59538311	0.3581877	1	0.72630738	0.53788181	0.333880598	1	0.74839344	0.709577147	0.53130364
Snail/GAPDH/Control	1	0.7127981	0.644994	0.280951	1	0.80918942	0.50366663	0.325792856	1	0.77246581	0.618238082	0.23409226

Note: (E)and **(F)** represent the raw data from three independent Western blot replicates **(e)** and the statistical analysis of the three independent replicates **(f)** corresponding to panels **E** and **(F** in Figure 8, respectively. The red boxes in panel **(e)** indicate the original Western blot bands used in Figure 8E, and the red-marked values in panel **(f)** are the data used for the statistical graph in Figure 8F.

(G) Supplementary Material Panels (G), (H), (I), (J), (K), (L)present the original Western blot (WB) bands and corresponding quantitative data in Siha cells.
Rawdataofwesternblot (g) andstatisticalanalysis (h)
Raw data of three independent replicates and statistical analysis of Western blot corresponding to A and B in Supplementary Figure4

(g)

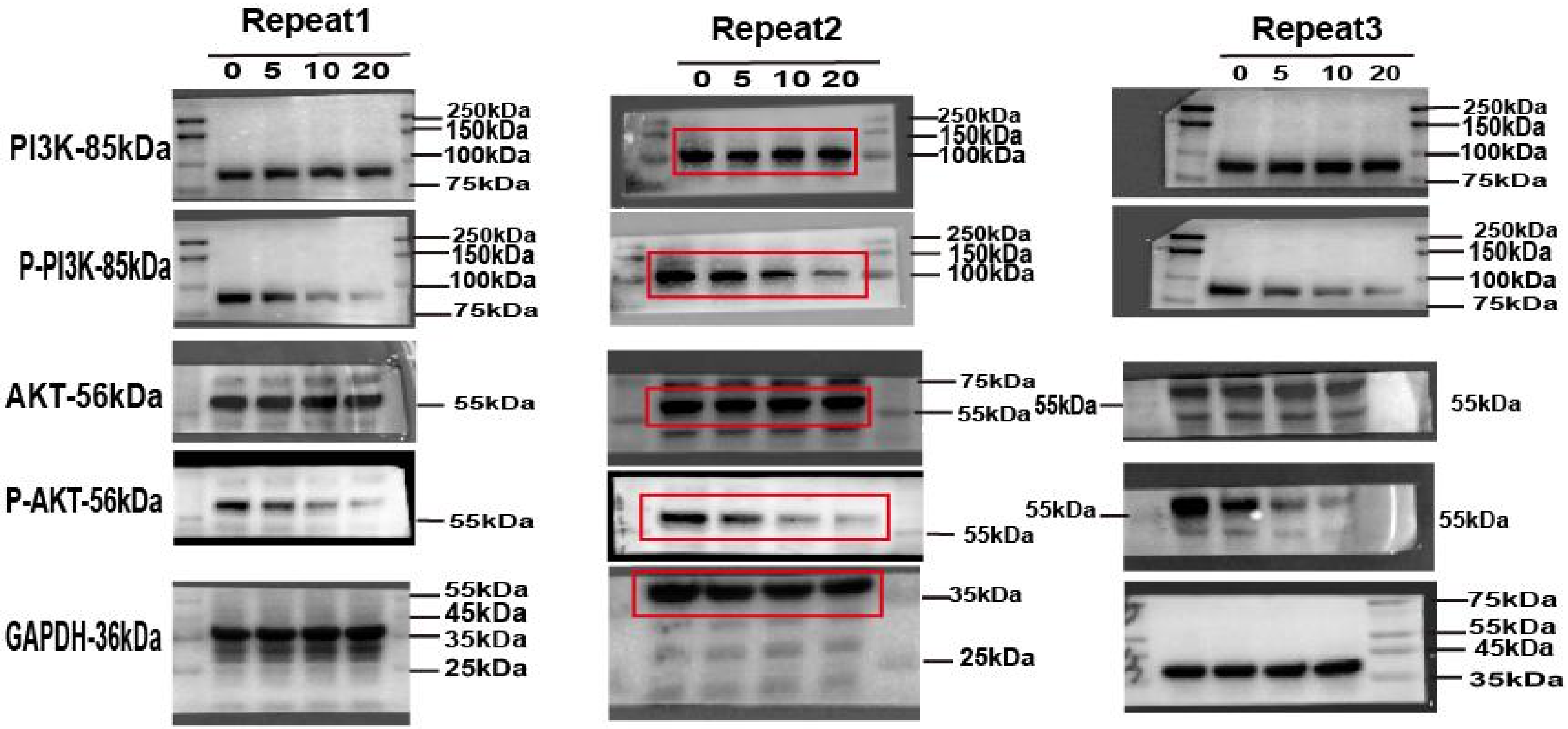

(H)

(h) Statistic alanalysis of data was used ImageJ

Oridonin (μmol/L)	Repeat1				Repeat2				Repeat3			
	0	5	10	20	0	5	10	20	0	5	10	20
PI3K	36862.903	35929.995	39420.48	31625.83	42232	39961.388	37523.359	34445.095	40471.681	40431.016	39550.167	39959.066
P-PI3K	42605.317	32456.53	20543.83	9469.539	40944	30303.61	22021.539	13100.075	46542.844	29568.995	17822.702	10013.569
P-PI3K/PI3K/Control	1	0.7855016	0.450905	0.259067	1	0.782179005	0.605338519	0.39228233	1	0.635945992	0.391853337	0.2179074
AKT	37735.924	38238.974	39992.46	35906.39	32693	37195.045	31288.823	31701.803	36869.752	37758.61	38537.803	30060.702
P-AKT	37192.874	29514.681	16065.8	7258.146	31611	23355.167	9939.66	5992.648	37231.288	24346.359	9554.439	5724.912
P-AKT/AKT/Control	1	0.7831178	0.407586	0.205092	1	0.649397094	0.328544919	0.19550022	1	0.638528342	0.245516337	0.1885957

Note: (G) and **(H)** represent the raw data from three independent Western blot replicates **(g)** and the statistical analysis of the three independent replicates **(h)** corresponding to panels **A** in **Supplementary Figure4**, respectively. The red boxes in panel **(g)** indicate the original Western blot bands used in A **Supplementary Figure4**, and the red-marked values in panel **(h)** are the data used for the statistical graph **B** in **Supplementary Figure4.**

.

(I) Supplementary Rawdataofwesternblot (i) andstatisticalanalysis (j)
Raw data of three independent replicates and statistical analysis of Western blot corresponding toC andD in Supplementary Figure4

(i)

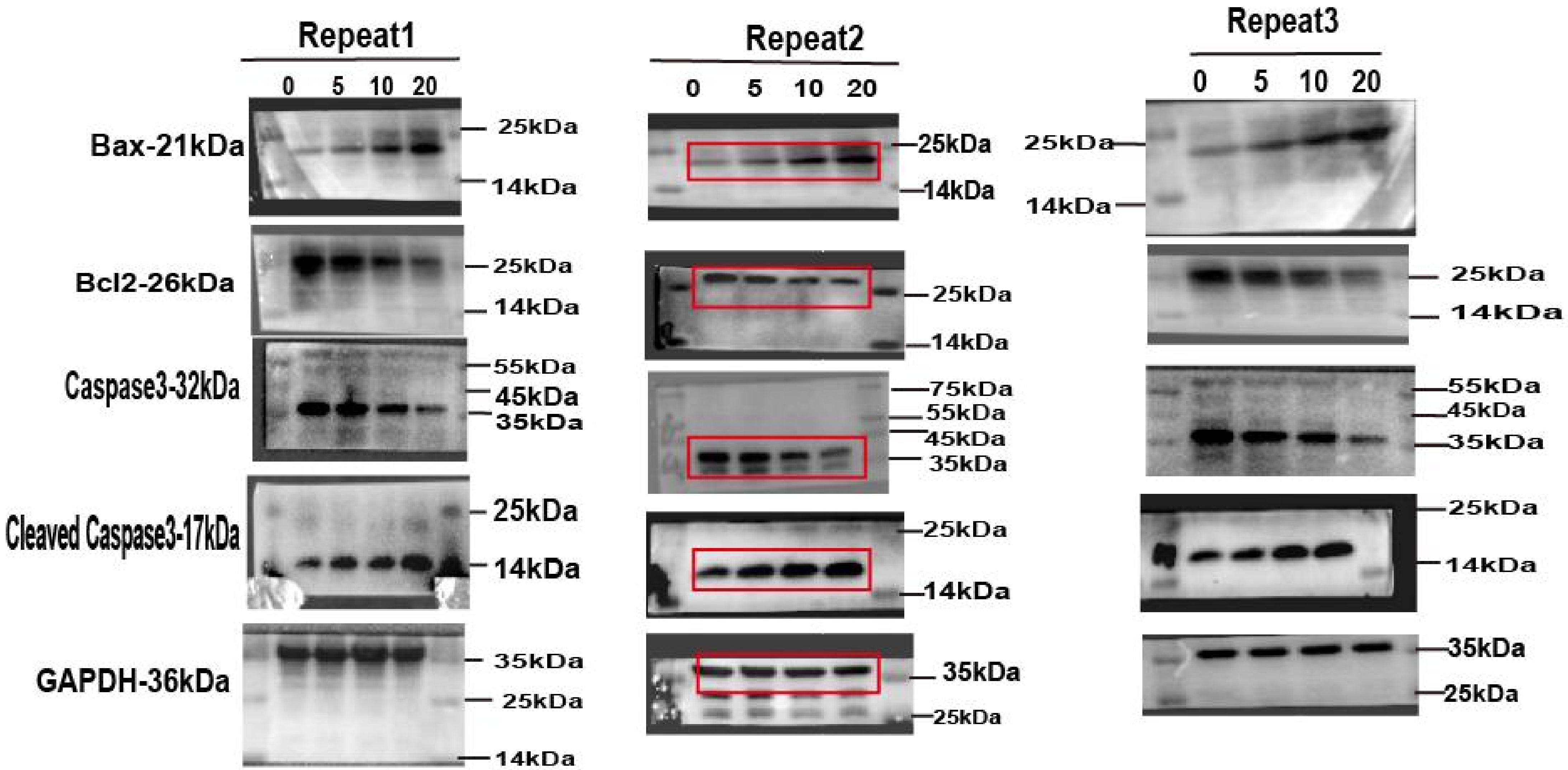

(J)

(j) **Statistic alanalysis of data was used ImageJ**

Oridonin (μmol/L)	Repeat1				Repeat2				Repeat3			
	0	5	10	20	0	5	10	20	0	5	10	20
Bax	16880.359	23796.459	25189.53	33568.066	23156.752	29848.995	35873.388	30404.167	15599.167	19373.581	32996.409	42785.803
Bcl-2	49026.773	34985.016	29306.602	20460.924	33998.652	30033.43	17072.995	9941.853	36700.631	33172.338	23672.095	20788.66
Bax/Bcl-2/Control	1	0.5061953	0.40058416	0.20986852	1	0.685316467	0.3241554	0.22271536	1	0.72776984	0.30492841	0.20651647
GAPDH	32488.489	32816.56	33443.024	33721.681	38077.681	39078.166	39428.439	39058.874	32468.489	33807.154	32463.027	33552.596
Caspase3	42998.945	27732.095	18655.066	11629.853	37787.238	28848.53	22231.137	7884.004	35392.924	30877.995	24655.288	11011.803
Caspase3/GAPDH/Control	1	0.63850064	0.4214664	0.26057739	1	0.743900511	0.56816887	0.20340071	1	0.83788828	0.69673351	0.30107725
Cleaved Caspase3	14279.61	24944.288	35481.995	43389.48	13635.631	26247.116	33325.116	39513.045	19588.167	33322.238	42149.045	49966.137
Cleaved Caspase3 /GAPDH//Control	1	1.72938316	2.41387989	2.92744266	1	1.875610556	2.36024637	2.82498425	1	1.63378096	2.15212259	2.46841358

Note:(I) and **(J)**represent the raw data from three independent Western blot replicates **(i)** and the statistical analysis of the three independent replicates **(j)** corresponding to panels **C** in **Supplementary Figure4**, respectively. The red boxes in panel **(i)** indicate the original Western blot bands used in **C Supplementary Figure4**, and the red-marked values in panel **(j)** are the data used for the statistical graph **D** in **Supplementary Figure4.**

(K) Supplementary Rawdataofwesternblot（k）andstatisticalanalysis（l）
Raw data of three independent replicates and statistical analysis of Western blot corresponding to E and F in Supplementary Figure4

(k)

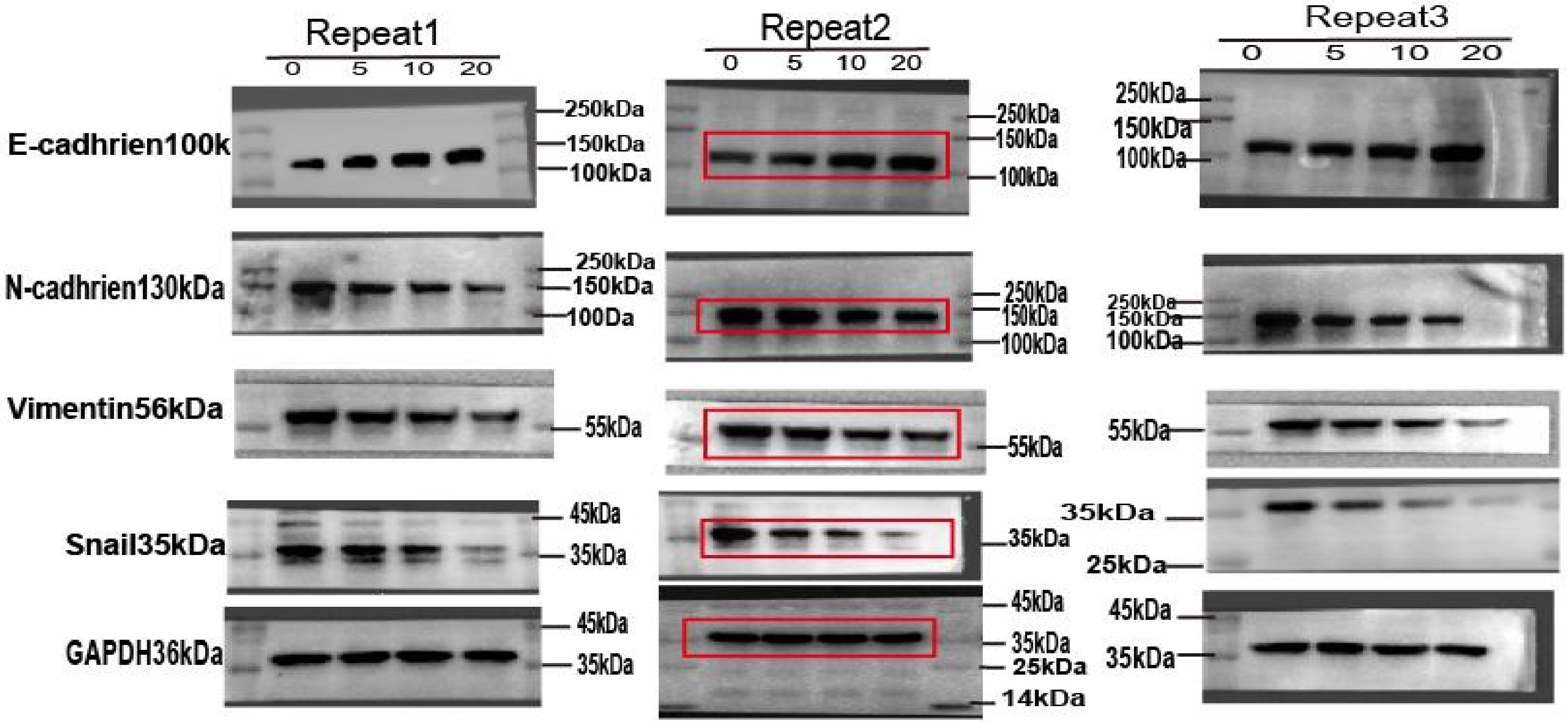

(L)

(l) Statistic alanalysis of data was used ImageJ

Oridonin (μmol/L)	Repeat1				Repeat2				Repeat3			
	0	5	10	20	0	5	10	20	0	5	10	20
E-cadhrien	23459.123	35167.539	46366.711	49105.539	18950.853	26992.025	37199.853	46213.782	26607.13	38994.439	45795.253	56989.367
N-cadhrien	39540.234	28923.023	26986.231	18311.981	36840.874	29698.982	23952.652	18650.165	39850.98	28997.338	23988.933	17786.121
Vimentin	39826.332	32595.954	26604.934	18032.924	36982.652	28983.168	21897.892	16304.853	39960.13	31922.623	24365.154	17986.62
Snail	39036.989	32385.212	28259.322	12398.398	39895.324	31601.203	26863.298	11701.256	38210.63	27902.933	21985.905	10236.66
GAPDH	36821.208	37148.124	38318.203	35246.136	45974.918	45992.238	46315.236	46169.288	46589.14	45123.945	46392.398	45302.156
E-cadhrien/GAPDH/Control	1	1.48590605	1.899273098	2.18678069	1	1.423780777	1.948540975	2.428345821	1	1.513151249	1.72846411	2.202732132
N-cadhrien/GAPDH/Control	1	0.7250461	0.65583697	0.4838187	1	0.80583859	0.64538782	0.50410446	1	0.75127114	0.604518709	0.45899506
Vimentin/GAPDH/Control	1	0.8112497	0.64192572	0.4730231	1	0.78340118	0.58776178	0.439022343	1	0.82480119	0.612322349	0.46290136
Snail/GAPDH/Control	1	0.8223024	0.69563003	0.3317995	1	0.79180463	0.66839688	0.292064163	1	0.75395124	0.577827213	0.27551159

Note:(K) and **(L)** represent the raw data from three independent Western blot replicates **(k)** and the statistical analysis of the three independent replicates **(l)** corresponding to panels **E** in **Supplementary Figure4**, respectively. The red boxes in panel **(k)** indicate the original Western blot bands used in **E Supplementary Figure4**, and the red-marked values in panel **(l)** are the data used for the statistical graph **F** in **Supplementary Figure4.**
