# Supplementary material for: Integrative network pharmacology, molecular dynamics simulation, and single-cell RNA sequencing strategies reveal the multi-target mechanisms of oridonin against cervical cancer
Source: Front Pharmacol. 2026 Jun 17;17:1788376. doi: 10.3389/fphar.2026.1788376 (PMC13318904; doi:10.3389/fphar.2026.1788376)
Supplement: Supplementary file 5 [file Image1.pdf]

Integrative network pharmacology, molecular dynamics simulation, and single-cell RNA sequencing strategies reveal the multi-target mechanisms of oridonin against cervical cancer

Min Xu†[1,2,3], TaoLu†[1,2,4], Qing LV[1,2],Kai Ju Mo[1,2], Yan-jie Liu*[1,2,5,]

*** Correspondence:** Dr.Yan-jie Liu

Corresponding Author： Department of Pathology in the Affiliated Hospital, Guizhou Medical University, Guiyang 550004, China. Address:No. 9 Beijing Road, Guiyang City, Guizhou Province,email@uni.edu： liuyanjie@gmc.edu.cn

Supplementary Figures

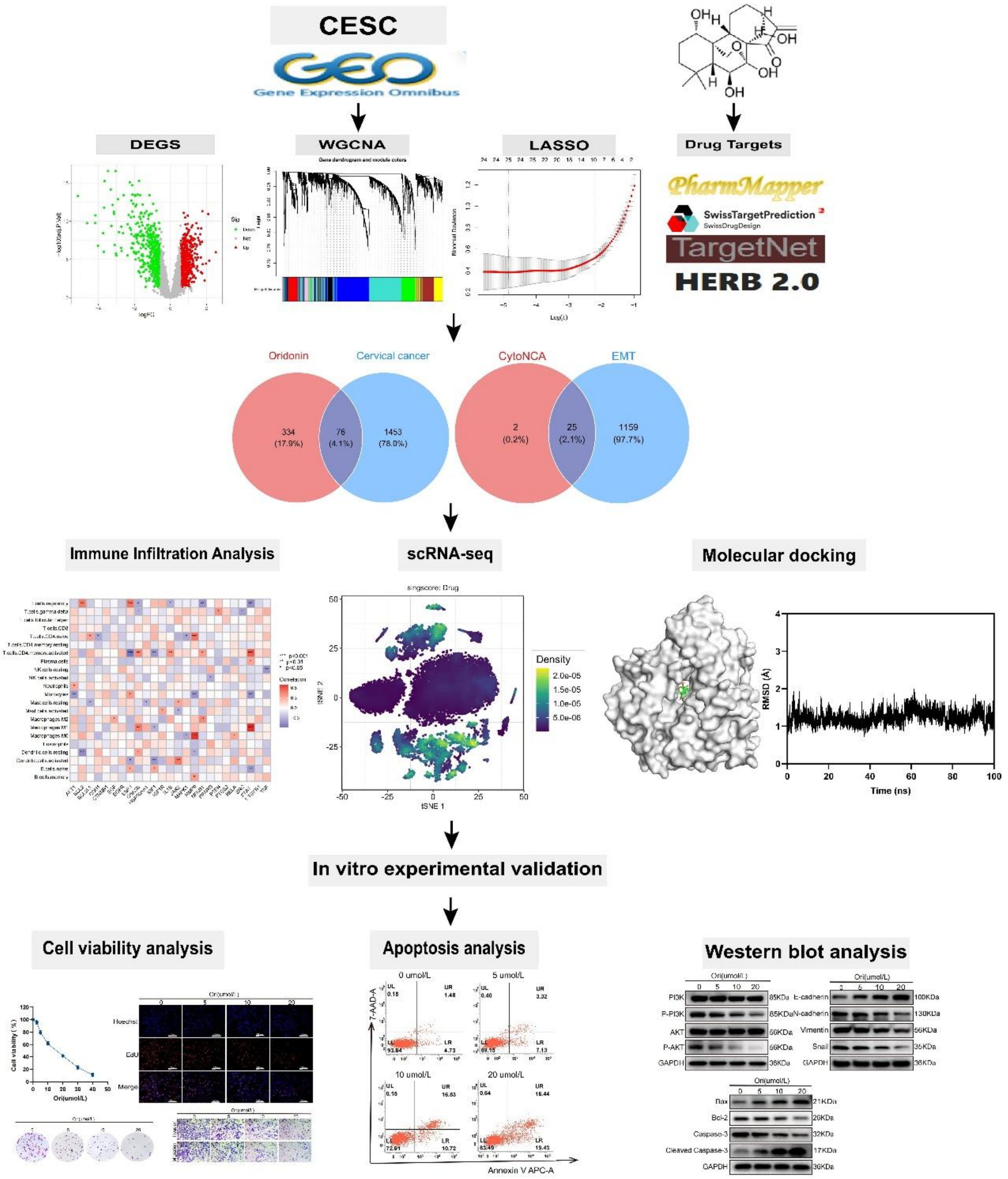

Supplementary Figure 1 **Flowchart of overall met hodology used to predict effect of Ori for CESC.**

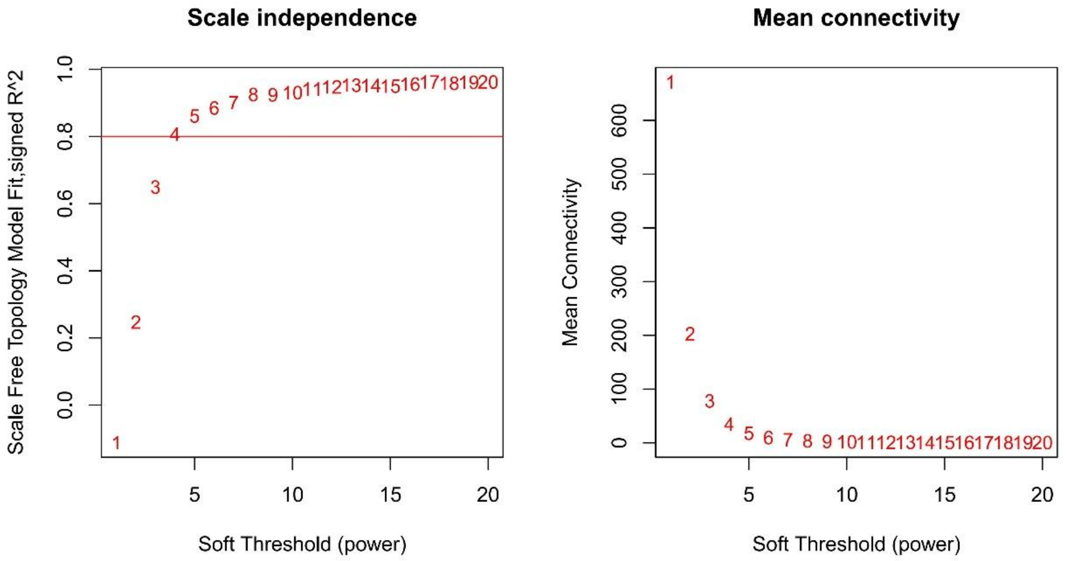

Supplementary Figure 2.Selection of the soft-thresholding power in WGCNA. Determination of the soft-thresholding power for WGCNA. After evaluating power values from 1 to 20, β = 5 was selected as it met the scale-free topology criterion ($R^2 \geq 0.8$).

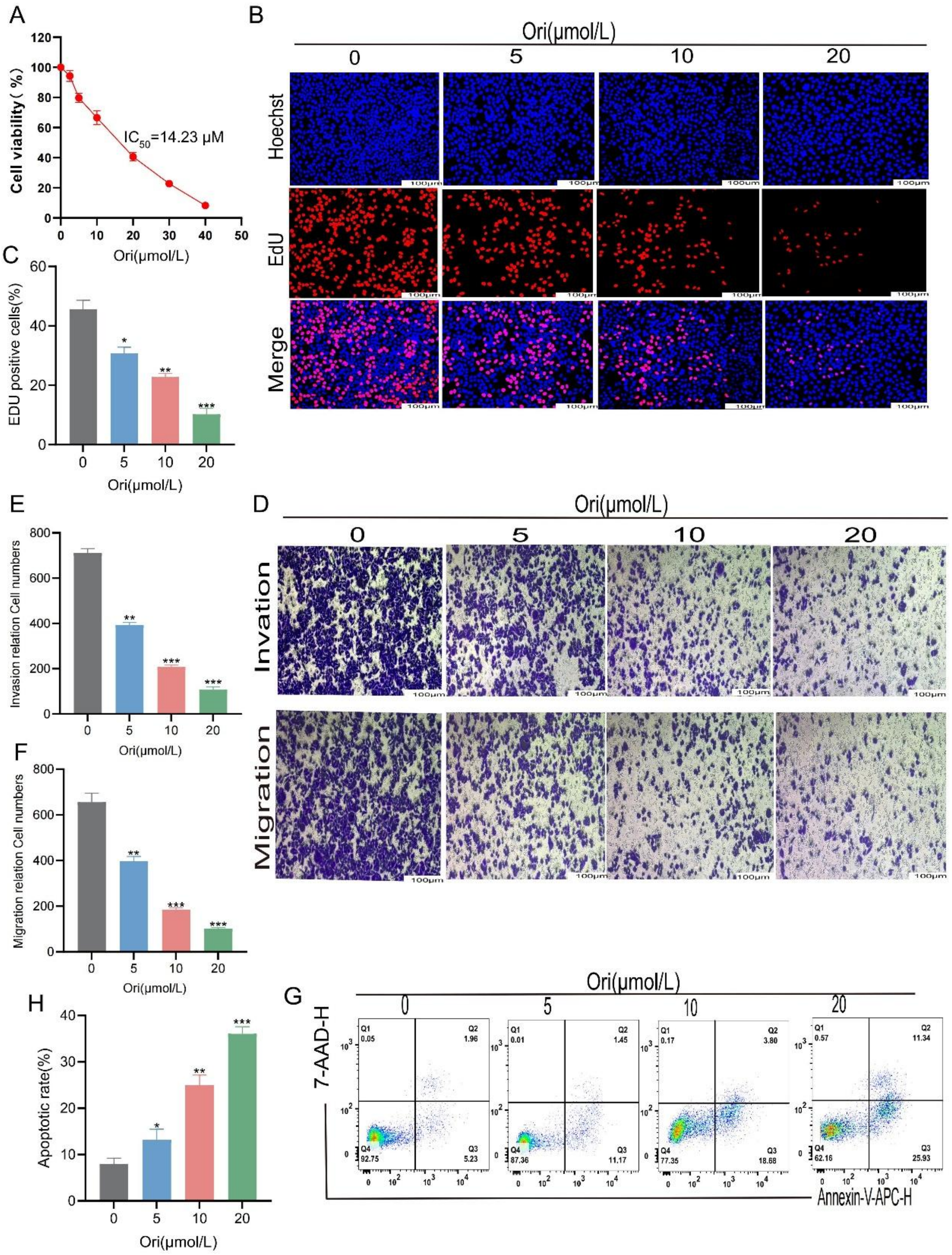

Supplementary Figure 3. Ori can inhibit the proliferation, migration and invasion of Siha cells and induce cell apoptosis. (A)Cell viability of Siha cells after 24 h treatment with different concentrations of Ori measured by the CCK-8 assay. (B)EdU staining results after 24 h of Ori treatment: active DNA is labeled in red, and nuclei are stained with Hoechst (blue). (C)Statistical analysis of the percentage of EdU-positive cells in each group. (D)Effects of Ori on migration and invasion of Siha cells evaluated by Transwell assays. (E,F)Statistical analysis of Transwell assays. (G)Flow cytometric analysis of apoptosis in Siha cells after 24 h of Ori treatment (Annexin V/7-AAD staining). （H） Statistical analysis of

apoptosis rates in each group. Data are presented as the mean ± SD from *n*=3 independent biological replicates. Statistical significance was determined using one-way ANOVA followed by Tukey's post-hoc test . Scale bar = 100 μm ,$*p < 0.05$, $**p < 0.01$, $***p < 0.001$.

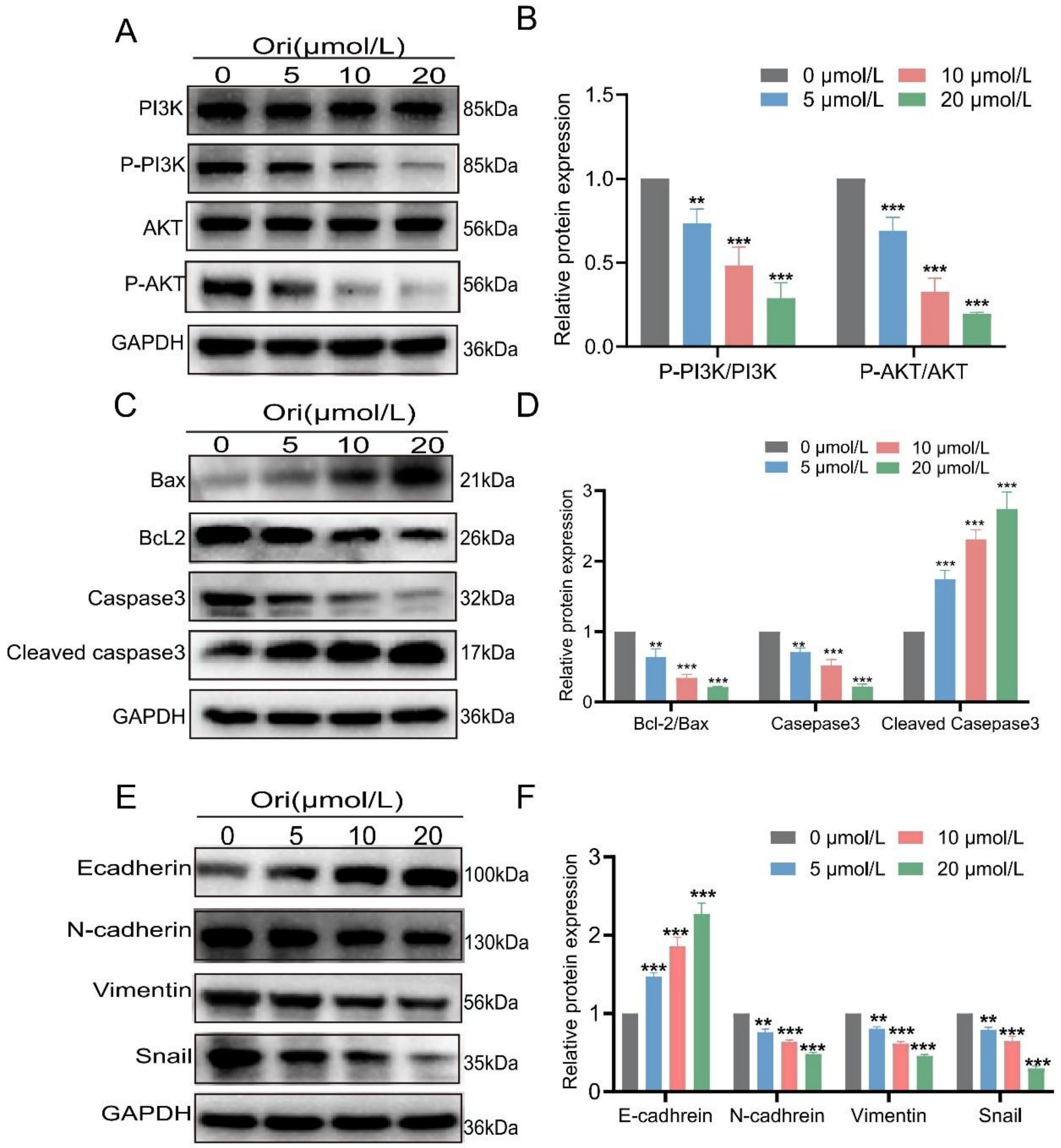

Supplementary Figure 4 Regulatory effects of Ori on apoptosis, PI3K/AKT signaling pathway and EMT-related proteins in Siha cells. (A)Western blot results of PI3K, p-PI3K, AKT and p-AKT proteins in Siha cells treated with different concentrations of Ori. (B)Quantitative analysis of the relative expression levels of p-PI3K/PI3K and p-AKT/AKT. (C)Expression levels of apoptosis-related proteins BAX, BCL-2, caspase-3 and cleaved caspase-3. (D)Quantitative,analysis of the BCL-2/BAX ratio and the expression levels of caspase-3 and cleaved caspase-3. (E)Western blot results of EMT marker proteins E-cad herin, N-cad herin,vimentin and Snail. (F)Quantitative analysis of the relative expression levels of E-cad herin, N-cad herin, vimentin and Snail.

Data are presented as the mean ± SD from n=3 independent biological replicates. Statistical significance was determined using one-way ANOVA followed by Tukey's post-hoc test. *$p < 0.05$, **$p < 0.01$, ***$p < 0.001$.
